# Genome-Scale Metabolic Reconstruction of *Acetobacter pasteurianus* 386B, a Candidate Functional Starter Culture for Cocoa Bean Fermentation

**DOI:** 10.3389/fmicb.2019.02801

**Published:** 2019-12-05

**Authors:** Rudy Pelicaen, Didier Gonze, Bas Teusink, Luc De Vuyst, Stefan Weckx

**Affiliations:** ^1^Research Group of Industrial Microbiology and Food Biotechnology (IMDO), Faculty of Sciences and Bioengineering Sciences, Vrije Universiteit Brussel (VUB), Brussels, Belgium; ^2^(IB)^2^ - Interuniversity Institute of Bioinformatics in Brussels (ULB-VUB), Brussels, Belgium; ^3^Unité de Chronobiologie Théorique, Service de Chimie Physique, Faculté des Sciences, Université Libre de Bruxelles (ULB), Brussels, Belgium; ^4^Systems Bioinformatics, Vrije Universiteit Amsterdam, Amsterdam, Netherlands

**Keywords:** acetic acid bacteria, *Acetobacter pasteurianus*, cocoa bean fermentation process, genome-scale metabolic model, genome annotation

## Abstract

*Acetobacter pasteurianus* 386B is a candidate functional starter culture for the cocoa bean fermentation process. To allow *in silico* simulations of its related metabolism in response to different environmental conditions, a genome-scale metabolic model for *A. pasteurianus* 386B was reconstructed. This is the first genome-scale metabolic model reconstruction for a member of the genus *Acetobacter*. The metabolic network reconstruction process was based on extensive genome re-annotation and comparative genomics analyses. The information content related to the functional annotation of metabolic enzymes and transporters was placed in a metabolic context by exploring and curating a Pathway/Genome Database of *A. pasteurianus* 386B using the Pathway Tools software. Metabolic reactions and curated gene-protein-reaction associations were bundled into a genome-scale metabolic model of *A. pasteurianus* 386B, named iAp386B454, containing 454 genes, 322 reactions, and 296 metabolites embedded in two cellular compartments. The reconstructed model was validated by performing growth experiments in a defined medium, which revealed that lactic acid as the sole carbon source could sustain growth of this strain. Further, the reconstruction of the *A. pasteurianus* 386B genome-scale metabolic model revealed knowledge gaps concerning the metabolism of this strain, especially related to the biosynthesis of its cell envelope and the presence or absence of metabolite transporters.

## Introduction

Acetic acid bacteria (AAB) are obligately aerobic bacteria that play an important role in several food fermentation processes, such as vinegar production and cocoa pulp-bean mass fermentation, although they are undesired in wine, cider, and most beer fermentation processes (De Roos and De Vuyst, 2018). AAB can be found in carbohydrate-rich environments, but also in acidic and alcoholic niches (Mamlouk and Gullo, 2013). Their typical metabolic trait is the incomplete oxidation of substrates using a specific respiratory chain. That way, ethanol is converted into acetic acid. Depending on species and strain, also sugar alcohols can be converted, such as glycerol into dihydroxyacetone, D-mannitol into D-fructose, or D-sorbitol into L-sorbose. Even organic acids can be oxidized, such as acetic acid into carbon dioxide and water, which is in fact an overoxidation (Mamlouk and Gullo, 2013).

The cocoa bean fermentation process is fundamental to obtain well-fermented dry cocoa beans, from which cocoa products, such as chocolate, can be made (De Vuyst and Weckx, 2016). Next to two other groups of key microorganisms present during cocoa bean fermentation processes, namely yeasts and lactic acid bacteria, AAB are crucial to produce the necessary acetic acid that contributes to the death of the cocoa bean embryo and subsequent formation of cocoa flavor precursors inside the beans (De Vuyst and Weckx, 2016).

*Acetobacter pasteurianus* is an AAB species that has been isolated consistently from cocoa bean fermentation processes around the world (Camu et al., 2007; Lefeber et al., 2011; Meersman et al., 2013; Papalexandratou et al., 2013; Miescher Schwenninger et al., 2016; Visintin et al., 2016; Ozturk and Young, 2017). Its apparent adaptation to this fermentation process has been explained by its high ethanol, acid, and heat tolerance (Camu et al., 2007; Illeghems et al., 2013). The strain *A. pasteurianus* 386B has been isolated from a spontaneous cocoa bean fermentation process performed in Ghana (Camu et al., 2007), and has subsequently been selected as a candidate functional starter culture because of its rapid co-consumption of ethanol and lactate, the production of acetate and acetoin, and the achievement of high cell densities upon fermentation (Lefeber et al., 2010; Moens et al., 2014). To gain more insight into its metabolic potential and niche adaptations, the *A. pasteurianus* 386B genome has been sequenced and annotated, resulting in a genome encompassing a 2.8-Mb chromosome and seven plasmids with 2,875 protein-encoding genes (Illeghems et al., 2013). Several characteristic metabolic pathways of AAB have been identified in the genome of this strain, for example an incomplete Embden-Meyerhof-Parnas (EMP) pathway, a modified tricarboxylic acid (TCA) cycle, and a truncated respiratory chain. Whereas genome annotation combined with *in vitro* experiments (Lefeber et al., 2010; Illeghems et al., 2013; Moens et al., 2014), have led to a first general view on the central carbon metabolism of *A. pasteurianus* 386B, the macromolecule biosynthesis pathways of this strain in particular and of AAB in general are still largely unknown.

Defined media have been used successfully in different microbiological studies to reveal the specific growth requirements of microorganisms and eventual metabolic adaptations to changes in medium composition, unraveling their biosynthesis capacities (Verduyn et al., 1992; van Niel and Hahn-Hägerdal, 1999; Richards et al., 2014). A complementary strategy is to reconstruct the metabolic network of a particular microorganism *in silico* and assess the accuracy of this reconstruction by comparing the outcome of *in silico* and *in vitro* growth experiments in defined media (Teusink et al., 2005). This metabolic network reconstruction process is based on cataloging a set of functionally annotated enzymes and transporters encoded in the genome and coupling them to their respective biochemical reactions *via* gene-protein-reaction (GPR) associations (Francke et al., 2005; Pitkänen et al., 2010; Thiele and Palsson, 2010). In addition, a (species-specific) biomass reaction has to be added to the model to be able to simulate the biomass production, which is used as a proxy for the specific growth rate of the bacterial cell population (Feist and Palsson, 2010).

So far, genome-scale metabolic models (GEMs) have been reconstructed for many organisms, ranging from bacteria and archaea to fungi, plants, and even human cell lines (Ruppin et al., 2010; Yilmaz and Walhout, 2017). For AAB, the only GEMs that are currently available are those for *Gluconobacter oxydans* 621H, an industrially important bacterium due to its property of oxidizing a wide range of carbohydrates, and for *Komagataeibacter nataicola* RZS01, a bacterial cellulose producer (Wu et al., 2014; Zhang et al., 2017). The current study aims to perform a reconstruction of a GEM for *A. pasteurianus* 386B, which is the first GEM for a species of the genus *Acetobacter*. Enzymes and transporters related to the consumption of substrates present in the cocoa pulp-bean mass and metabolites produced thereof were specifically targeted. It is expected that this GEM will be useful to perform *in silico* metabolic flux simulations of *A. pasteurianus* 386B in response to different environmental conditions to improve the cocoa bean fermentation process.

## Materials and Methods

### *Acetobacter pasteurianus* 386B Genome Re-annotation and *in silico* Genome-Scale Metabolic Reconstruction

**Figure 1:**
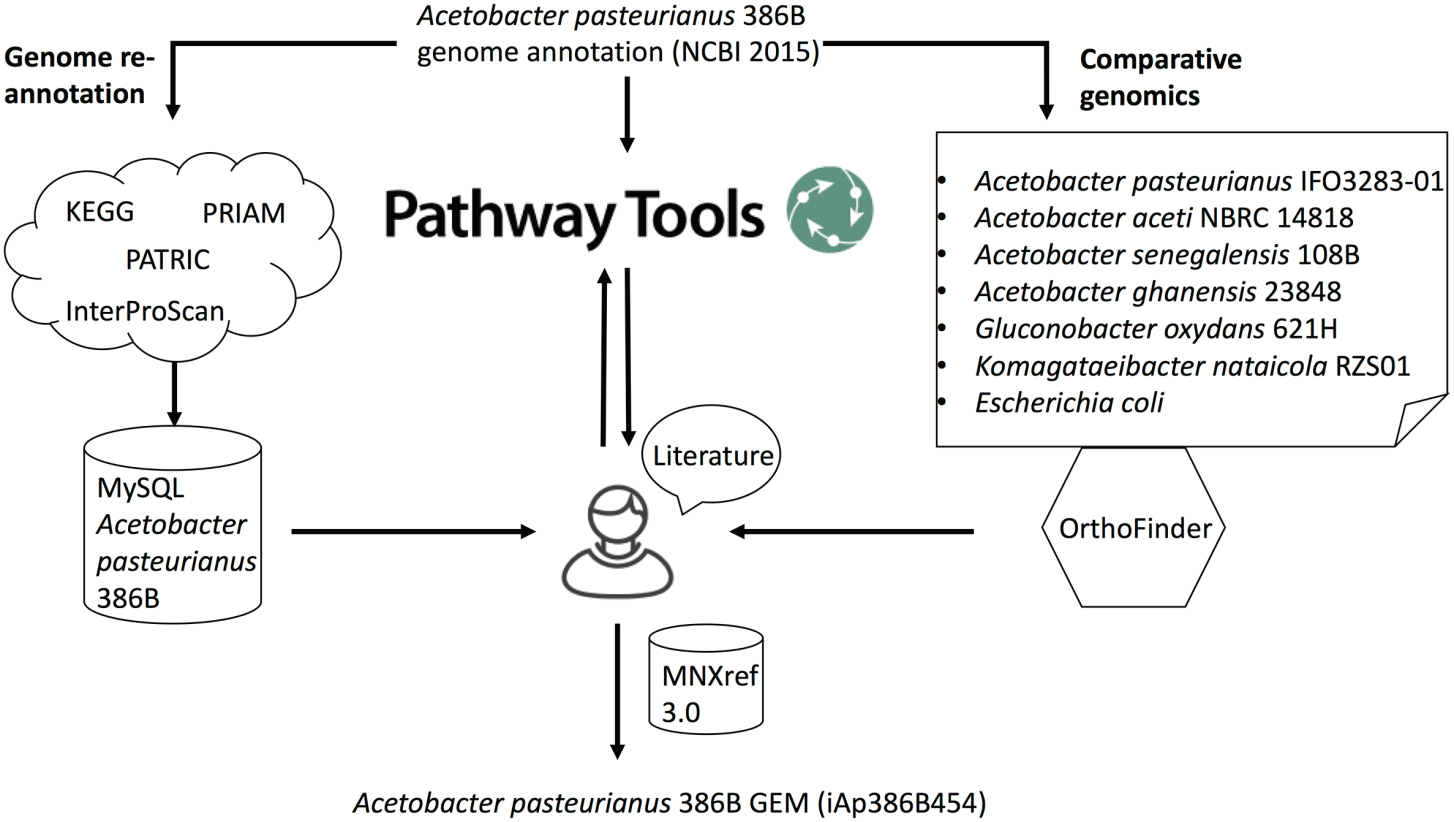
Schematic overview of the metabolic network reconstruction process. Genome re-annotation of *A. pasteurianus* 386B was performed using a combination of several databases (*e.g.* KEGG, PATRIC) and tools (e.g., PRIAM, InterProScan). Functional annotations were stored in a MySQL database. Comparative genomics using OrthoFinder allowed to identify orthogroups of protein sequences between *A. pasteurianus* 386B and other phylogenetically related bacterial species. Pathway Tools was used to predict metabolic pathways as well as the reactions and gene-protein-reaction associations that constitute these pathways. This information was stored in a Pathway/Genome database (PGDB). Edges entering the human curator node represent the information sources that were used to guide manual curation. Using additional information from the literature, the *A. pasteurianus* 386B PGDB was curated and its information transferred to a new genome-scale metabolic model of *A. pasteurianus* 386B, iAp386B454, using the MNXref 3.0 namespace of reactions and metabolites.

The complete genome of *A. pasteurianus* 386B was sequenced and annotated previously, using a local installation of the bacterial genome annotation system GenDB v2.2 (Meyer, 2003; Illeghems et al., 2013). Since then, different genome annotations of this strain became publicly available in different databases. To perform a thorough re-annotation of the *A. pasteurianus* 386B genome and to be able to assess differences between the annotation sources, the annotation data were collected in a MySQL database that was built in-house (Figure 1). Publicly available genome annotation sources included the Carbohydrate-Active enZYmes database (CAZy; Lombard et al., 2014), the Kyoto Encyclopedia of Genes and Genomes (KEGG; Kanehisa et al., 2017), the Integrated Microbial Genomes platform of the Joint Genome Institute (JGI IMG; Markowitz et al., 2012), the RefSeq database of the National Center for Biotechnology Information (NCBI; O’Leary et al., 2016), the Pathosystems Resource Integration Center database (PATRIC; Wattam et al., 2017), the proGenomes database (Mende et al., 2017), and TransportDB (Elbourne et al., 2017). The latter database relies on the TC system for annotation, providing a defined ontology to describe transporter functions in analogy to the EC system for enzyme annotation (Saier et al., 2016). Two annotation versions were used in the case of the NCBI RefSeq annotation source, one published in April 2015 (further referred to as NCBI 2015) and one published in April 2017 (further referred as NCBI 2017). Differences in these genome annotations reflect improvements made in the NCBI prokaryotic genome annotation pipeline (Tatusova et al., 2016). Furthermore, the *A. pasteurianus* 386B genome was re-annotated in-house, using the subcellular localisation predictor CELLO (Yu et al., 2004), eggNOG-mapper (Huerta-Cepas et al., 2017), the enzyme annotation tool PRIAM (Claudel-Renard, 2003), and the tools embedded in InterProScan 5.22-61.0 (Figure 1; Jones et al., 2014).

As a reference set of predicted protein-encoding genes, the NCBI 2015 genome annotation was used, containing 2,854 protein-encoding genes, as it is the same that was used by SRI International to reconstruct the *A. pasteurianus* 386B Pathway/Genome Database (PGDB) available on BioCyc (Caspi et al., 2016). Subsequently, for each protein-encoding gene, functional annotations from the different annotation sources were added to the MySQL database. In addition, the *A. pasteurianus* 386B PGDB was manually curated in the Pathway Tools software, assisted by different automatic refinements using the Pathologic tool inside this software (Figure 1; Karp et al., 2016). Features associated to a predicted MetaCyc pathway (Caspi et al., 2016), such as the taxonomic range, pathway score, pathway variants and pathway description, were manually assessed and compared to information available in the literature to decide whether the pathway in question should be omitted.

Also, comparative genomics was performed using OrthoFinder, allowing to predict orthogroups from protein-encoding genes in whole genomes (Figure 1; Emms and Kelly, 2015). Protein sequences of a selection of bacterial species obtained from NCBI RefSeq were compared, namely *A. pasteurianus* 386B, *A. pasteurianus* IFO3283-01, *A. aceti* NBRC 14818, *A. senegalensis* 108B, *A. ghanensis* LMG 23848^T^, *Gluconobacter oxydans* 621H, *Komagataeibacter nataicola* RZS01, and *Escherichia coli* str. K-12 substr. MG1655 (further referred to as *E. coli*). For the latter three bacterial strains, genome-scale metabolic models have been reconstructed before (Orth et al., 2011; Wu et al., 2014; Zhang et al., 2017).

Finally, based on the information contained in the in-house built MySQL database, the *A. pasteurianus* 386B PGDB, and the outcome of the comparative genomics analysis, the *A. pasteurianus* 386B GEM was reconstructed and manually curated using the MNXref 3.0 namespace as a biochemical reaction repository (Figure 1; Bernard et al., 2014; Moretti et al., 2016). Characterized enzymes and associated protein sequences available in the literature were used to perform sequence alignment searches using blastp (Altschul et al., 1990). The best blast hits in the *A. pasteurianus* 386B genome are indicated by their percentage similarity, percentage identity, and percentage coverage (Supplementary Table S1).

MetaCyc reaction components and GPR associations of biosynthesis pathways in the curated *A. pasteurianus* 386B PGDB were transferred to the *A. pasteurianus* 386B GEM by mapping the MetaCyc reaction components to MNXref 3.0 reaction identifiers. Reactions described in the literature but not present in the MNXref 3.0 reaction repository were manually added to the model. Enzyme names and enzyme commission (EC) numbers were assigned to each reaction, based on the ExplorEnz database (McDonald et al., 2009). The mass and charge balances were checked for all reactions in the resulting *A. pasteurianus* 386B GEM. The co-factor specificity of these reactions was manually curated, based on functional annotations and literature sources. Reaction reversibility constraints were manually curated, taking into account the directionality defined in the MetaCyc database, and to prevent reaction flux cycles from occurring in the GEM. The list of GPR associations was saved in a spreadsheet file (Supplementary File S2). The *A. pasteurianus* 386B GEM, named iAp386B454, was reconstructed and analyzed using the COBRAPy package version 0.11.3 (Ebrahim et al., 2013). Next, it was exported in the Systems Biology Markup Language (SBML) level 3 format (Supplementary File S3) and validated using the online SBML Validator (Bornstein et al., 2008). Reconstructed metabolic pathways were visualized using Escher (King et al., 2015).

### Evaluation of the Manual Curation Process and Comparison to Other Reconstructions

The presumed increase in functional annotation quality of the genes included in iAp386B454 was evaluated based on the EC numbers of enzyme-encoding genes, as the EC system provides a defined enzymatic reaction classification (McDonald et al., 2009). EC numbers of reactions associated to 304 selected genes in the *A. pasteurianus* 386B GEM were compared to the EC numbers of the genes in the original annotation sources stored in the MySQL database. A number of classes were defined based on the hierarchical EC system that expressed the agreement between the EC number of a gene in the GEM and that in the annotation source. Then, for each annotation source, genes were assigned to the predefined classes. Preference was given to assign a gene to a more precise class if at least one of the EC numbers of the gene in the annotation source did fulfill the requirement set upon that class. Enzyme systems, for which the reaction is catalyzed by a complex containing more than one enzyme (e.g., pyruvate dehydrogenase), were excluded from the analysis, as these reactions inherited different EC numbers from their gene constituents.

The manually reconstructed *A. pasteurianus* 386B GEM was compared to GEMs obtained from a number of automatic GEM reconstruction tools. These included the RAST and ModelSEED tools in the KBase software (Henry et al., 2010; Overbeek et al., 2014; Arkin et al., 2018), CarveMe (Machado et al., 2018), MetaNetX (Ganter et al., 2013), and Pathway Tools (Karp et al., 2016). The NCBI 2015 annotation version of *A. pasteurianus* 386B was used for the reconstructions, which were performed without reaction gap-filling. COBRAPy was used to parse the SBML files of the reconstructed GEMs, except for SBML files obtained from Pathway Tools, which were parsed using CBMPy version 0.7.19 (Olivier et al., 2005). Model properties were inferred from the reconstructions and compared to iAp386B454.

### Biomass Reaction

A biomass reaction was defined for *A. pasteurianus* 386B, using a combination of genomic and literature data (Supplementary File S4; Supplementary Figure S1). Protein, DNA, RNA, lipid, fatty acid, peptidoglycan, and carbohydrate mass fractions were taken from a GEM of *K. nataicola* RZS01 (Zhang et al., 2017). Stoichiometric coefficients of the biomass reaction were obtained by converting the mass fraction of the different macromolecules into a molar fraction, using their estimated molar masses. For each macromolecule, a separate biosynthesis reaction was defined. Stoichiometric coefficients of these reactions resulted from estimations of the molar fractions of the macromolecule building blocks. Molar fractions of amino acids for proteins and of nucleotides for DNA and RNA were estimated using the genome sequence of *A. pasteurianus* 386B, as proposed before (Thiele and Palsson, 2010). For DNA, molar fractions of building blocks were estimated using the G + C percentage of the genome (*in casu* 52.86%). For RNA, molar fractions of building blocks were estimated using their respective frequencies in rRNA-, tRNA-, and mRNA-encoding genes. RNA mass fractions were taken from *E. coli* (Milo and Phillips, 2016). The fatty acid and phospholipid compositions were taken from studies on related *Acetobacter* species (Yamada et al., 1981; Hanada et al., 2001). The average molar mass of a generic fatty acid was used to estimate the average molar mass of a generic phospholipid. Molar masses of macromolecule building blocks were queried in PubChem and ChEBI (Hastings et al., 2016; Kim et al., 2019). Energy requirements for protein, DNA, and RNA biosyntheses were taken from *E. coli* (Neidhardt et al., 1990). Genes were manually assigned to the different macromolecule biosynthesis reactions for cellular processes that were not explicitly included in the model, among which tRNA loading, protein elongation, replication, transcription, and translation.

### Growth Experiments

Growth experiments were performed with *A. pasteurianus* 386B in a modified defined medium (Verduyn et al., 1992). This medium contained (per liter): (NH_4_)_2_SO_4_, 5 g; KH_2_PO_4_, 1.375 g; MgSO_4_.7H_2_O, 0.5 g; EDTA, 15 mg; ZnSO_4_.7H_2_O, 4.5 mg; CoSO_4_.7H_2_O, 0.35 mg; MnCl_2_.4H_2_O, 1.0 mg; CuSO_4_.5H_2_O, 0.3 mg; CaCl_2_.2H_2_O, 4.5 mg; FeSO_4_.7H_2_O, 3.0 mg; MoO_3_, 0.24 mg; H_3_BO_3_, 1.0 mg; KI, 0.1 mg; and 30 mM phosphate buffer (pH 6.0). The pH of the medium was set to 5.0. A filter-sterilized vitamin mixture was added after heat sterilization (121°C, 2.1 bar, 20 min) of the medium. The final vitamin concentrations were (per liter): biotin, 0.0005 mg; calcium pantothenate, 0.01 mg; nicotinic acid, 0.01 mg; *myo*-inositol, 0.25 mg; thiamine-HCl, 0.01 mg; pyridoxine-HCl, 0.01 mg; and para-aminobenzoic acid, 0.002 mg. Eight different carbon sources (glucose, fructose, mannitol, citric acid, glycerol, lactic acid, ethanol, and sodium acetate) were used at a final concentration of 60 mM to assess if they could sustain growth. The pH of the citric acid, lactic acid (both by using NaOH to increase the pH), and sodium acetate (using HCl to decrease the pH) stock solutions was set to 5.0. To prepare the inocula, *A. pasteurianus* 386B was grown overnight at 30°C in 10 ml of an undefined medium (pH 5.5), which contained (per liter): lactic acid, 5 g; sodium acetate, 10 g; granulated yeast extract, 5 g; MgSO_4_.7H_2_O, 1 g; NH_4_H_2_PO_4_, 20 g; and K_2_HPO_4_, 10 g. The stirring rate was set to 160 rpm. The overnight culture was centrifuged (4,000 × *g*, 20 min, 4°C) and washed with a filter-sterilized saline solution (0.85%, m/v, NaCl). The cells were resuspended in the sterile saline solution and inoculated in 2 ml of the defined medium mentioned above at an optical density at 600 nm (OD_600_) of 0.01 in triplicate in test tubes with a total volume of 20 ml. *A. pasteurianus* 386B was allowed to grow at 30°C without shaking for 48 h as the test tubes contained only 2 ml culture medium, resulting in a relatively large surface to medium ratio. A threshold value for the OD_600_ of 0.1 was used to identify whether or not the strain had grown.

### Model Validation

*In silico* growth experiments were performed using flux balance analysis (FBA). With FBA, an optimization of the flux distribution of a GEM can be performed to maximize an objective function, typically the biomass reaction, thus predicting the specific growth rate of a bacterial cell population (Gottstein et al., 2016). FBA was performed with the *A. pasteurianus* 386B GEM, setting the biomass reaction as the objective of the simulation. Parameter values of this GEM included the consumption flux values of the different nutrients. Here, the consumption of ammonium as nitrogen source, sulfate as sulfur source, and phosphate as phosphate source were allowed without constraints. For aerobic respiration, an oxygen influx was allowed without constraint. D-glucose, D-mannitol, glycerol, D-lactate, ethanol, and acetate were tested as carbon sources separately to be able to compare the *in silico* results with the *in vitro* growth experimental data. The maximum exchange flux of the carbon source was set to 60 C-mmol/g_CDW_/h for each simulation. This value corresponded with a consumption of 10 mmol/g_CDW_/h of glucose for *E. coli* (Varma et al., 1993).

## Results and Discussion

### Genome Re-annotation

An extensive re-annotation of the genome of *A. pasteurianus* 386B was performed, based on a combination of several databases and tools, comparative genomics analyses, and manual curations using different software packages (Figure 1). Of a total of 2,875 protein-encoding genes in the original genome annotation of *A. pasteurianus* 386B (Illeghems et al., 2013), 2,854 genes (NCBI 2015) were re-annotated in the current study, of which 454 genes were included in the *A. pasteurianus* 386B GEM based on their involvement in the metabolic pathways discussed below. In what follows, metabolic reactions in different parts of the *A. pasteurianus* 386B metabolism are discussed, based on the evidence that the enzyme-encoding genes were present in the genome.

### Central Carbon Metabolism

**Figure 2:**
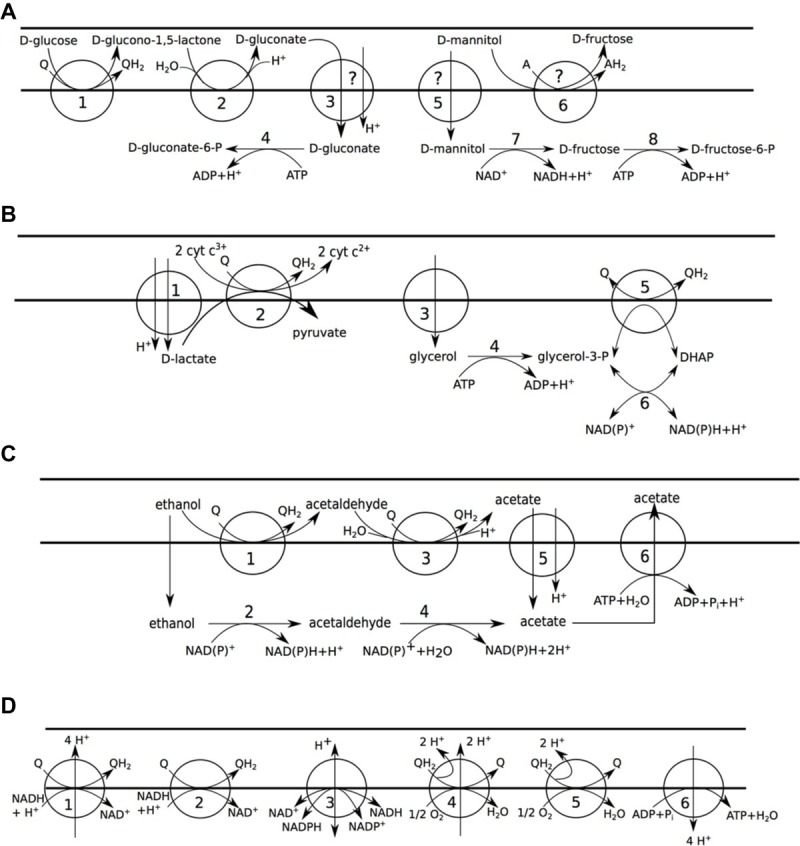
Carbon metabolism of *A. pasteurianus* 386B. Horizontal bars (from top to bottom) delineate the extracellular environment, the periplasm, and the cytosol, respectively. **(A)** Carbohydrate oxidation. (1) D-glucose:ubiquinone oxidoreductase (EC 1.1.5.2); (2) gluconolactonase (EC 3.1.1.17); (3) gluconate:H^+^ symport; (4) gluconokinase (EC 2.7.1.12); (5) mannitol permease; (6) D-sorbitol dehydrogenase (mann_ox_rxn); (7) mannitol 2-dehydrogenase (EC 1.1.1.67); (8) fructokinase (EC 2.7.1.4). **(B)** Lactate and glycerol oxidation. (1) lactate:H^+^ symporter; (2) D-lactate dehydrogenase (quinone) (EC 1.1.5.12), D-lactate dehydrogenase (cytochrome) (EC 1.1.2.4); (3) glycerol facilitator; (4) glycerol kinase (EC 2.7.1.30); (5) glycerol-3-phosphate dehydrogenase (EC 1.1.5.3); (6) glycerol-3-phosphate dehydrogenase [NAD(P)^+^] (EC 1.1.1.94). **(C)** Ethanol oxidation. (1) alcohol dehydrogenase (quinone) (EC 1.1.5.5); (2) alcohol dehydrogenase [NAD(P)^+^] (EC 1.1.1.1, 1.1.1.2); (3) aldehyde dehydrogenase (quinone) (EC 1.2.5.2); (4) aldehyde dehydrogenase [NAD(P)^+^] (EC 1.2.1.3, EC 1.2.1.4); (5) succinate-acetate:H^+^ symporter; (6) acetate ABC transporter. **(D)** Aerobic respiration. (1) NADH:ubiquinone reductase (H^+^-translocating) (EC 7.1.1.2); (2) NADH:ubiquinone reductase (non-electrogenic) (EC 1.6.5.9); (3) proton-translocating NAD(P)^+^ transhydrogenase (EC 7.1.1.1); (4) ubiquinol oxidase (H^+^-transporting) (EC 7.1.1.3); (5) ubiquinol oxidase (electrogenic, non-H^+^-transporting) (EC 7.1.1.7); (6) H^+^-transporting two-sector ATPase (EC 7.1.2.2).

The incomplete EMP pathway (Supplementary Figure S2) and the pentose phosphate pathway (Supplementary Figure S3) of *A. pasteurianus* 386B were reconstructed, as described previously (Illeghems et al., 2013). Periplasmic oxidation of D-glucose could be assigned to a pyrroloquinoline quinone (PQQ)-dependent glucose 1-dehydrogenase (EC 1.1.5.2) (Figure 2A). Based on the re-annotation effort of the current study, different gene candidates could be linked to this reaction (e.g., APA386B_2133, Supplementary Table S1), among which genes with a previously unknown function (Illeghems et al., 2013). No transporter could be identified for D-gluconate uptake. However, since the gene encoding cytoplasmic gluconokinase was present in the genome (locus tag APA386B_1158), the D-gluconate transport reaction was nonetheless added to the *A. pasteurianus* 386B GEM. Cytoplasmic oxidation of D-mannitol could be assigned to a cytoplasmic mannitol 2-dehydrogenase. However, the possibility of periplasmic oxidation of D-mannitol to D-fructose was included in the GEM as well, since extracellular fructose formation from mannitol has been reported previously (Moens et al., 2014).

A gene encoding a lactate permease (TC 2.A.14), more precisely a lactate:H^+^ symporter, was identified in the genome, which was homologous to the LctP transporter in *E. coli* (Dong et al., 1993; Núñez et al., 2002). Most probably *A. pasteurianus* 386B oxidizes D-lactate using a D-lactate dehydrogenase that is dependent on quinone (EC 1.1.5.12) or cytochrome *c* (EC 1.1.2.4), thus linking this reaction directly to the respiratory chain (Figure 2B). Overflow of lactate to acetoin has been found and metabolic flux analysis has revealed that acetoin is solely produced by decarboxylation of (2S)-2-acetolactate (Adler et al., 2014; Moens et al., 2014). Therefore, this metabolic route, for which the genes were present, and containing the enzymatic reactions of acetolactate synthase (EC 2.2.1.6) and acetolactate decarboxylase (EC 4.1.1.5), was added to the *A. pasteurianus* 386B GEM (Supplementary Figure S4). Finally, an acetoin dehydrogenase complex (EC 2.3.1.190) was identified that could be involved in the oxidation of acetoin. A glycerol uptake transporter (TC 1.A.8) was identified in the genome that was homologous to the GlpF transporter of *E. coli* (Fu et al., 2000). Similarly as for lactate, oxidation of glycerol could feed electrons to the respiratory chain (Figure 2B); alternatively, glycerol may be used for glycerolipid biosynthesis.

*A. pasteurianus* 386B could oxidize ethanol to acetic acid in the periplasm by means of a membrane-bound PQQ-dependent alcohol dehydrogenase or in the cytoplasm by means of NAD(P)^+^-dependent dehydrogenases (Figure 2C). Periplasmic acetate could be imported by an acetate/succinate:H^+^ symporter, for which the encoding gene was identified (APA386B_1116) and which was homologous to the *E. coli* SatP transporter (Sá-Pessoa et al., 2013). Alternatively, excess cytoplasmic acetate may be exported by a primary active ABC transporter (TC 3.A; APA386B_103) (Nakano et al., 2006). A reaction cycle that regulates acetate overflow metabolism, involving acetic acid, acetyl phosphate, and acetyl-CoA, has been described in *E. coli* (De Mey et al., 2007; Valgepea et al., 2010). Putative orthologs for these enzymes were retrieved in *A. pasteurianus* 386B, namely genes encoding acetate kinase (EC 2.7.2.1), phosphate acetyltransferase (EC 2.3.1.8), and acetate CoA ligase (EC 6.2.1.1).

A major metabolic adaptation of acetic acid-resistant *Acetobacter* species is their modified tricarboxylic acid (TCA) cycle (Supplementary Figure S4), containing succinyl-CoA:acetate CoA-transferase (EC 2.8.3.18) and malate:quinone oxidoreductase (EC 1.1.5.4) (Mullins et al., 2008). Two enzymes involved in anaplerotic reactions were encoded in the genome, namely a reversible NAD^+^-dependent malic enzyme (EC 1.1.1.38), interconverting malate and pyruvate, and phosphoenolpyruvate carboxylase (EC 4.1.1.31) that could carboxylate phosphoenolpyruvate to oxaloacetate. However, in the NCBI 2017 genome annotation version of *A. pasteurianus* 386B, the latter enzyme-encoding gene was annotated as a pseudogene, containing a frameshift mutation.

### Aerobic Respiration

*A. pasteurianus* is an obligate aerobe that has evolved a truncated aerobic respiratory chain (Figure 2D). In *A. pasteurianus* 386B, a single gene cluster *cyaBACD* (APA386B_1578 – APA386B_1581) was found that encodes cytochrome *ba*_3_ ubiquinol oxidase (UOX *ba*_3_; EC 7.1.1.3). This heme-copper terminal oxidase probably contains a heme A moiety, since the genes encoding heme O (*ctaB*; APA386B_608) and heme A (*ctaA*; APA386B_1565) synthase, which are remnants of a cytochrome *c* oxidase gene cluster, have been shown to be still functional in *A. pasteurianus* (Matsutani et al., 2014). Additionally, *A. pasteurianus* 386B contained genes encoding two cytochrome *bd*-type oxidases, being cytochrome *bd* oxidase (APA386B_472 – APA386B_473) and its homolog, cyanide insensitive oxidase (CIO; APA386B_1010 – APA386B_1111). Since the reaction catalyzed by both of these terminal oxidases has the same stoichiometry, an identical reaction (EC 7.1.1.7) was added to the GEM. However, it has been shown that these enzymes have different kinetic parameters, indicating a physiological distinct role (Miura et al., 2013). Furthermore, two sets of genes for a cytochrome *bc*_1_ complex (E.C. 7.1.1.8) were found in the genome of *A. pasteurianus* 386B, as is also the case for *A. aceti* (Sakurai et al., 2011).

Next to ubiquinone, NAD(P)^+^ is involved in respiratory chain reactions. Two types of NADH:ubiquinone reductases were encoded in the *A. pasteurianus* 386B genome, namely one that is proton-translocating (EC 7.1.1.2) and one that is not (EC 1.6.5.9). Furthermore, a membrane-bound proton-translocating NAD(P)^+^ transhydrogenase (EC 7.1.1.1) was present in the genome. Finally, the genes encoding ATP synthase (EC 7.1.2.2) were present in two gene clusters (APA386B_1266 – APA386B_1270 and APA386B_1604 – APA386B_1608), which is also the case for *Rhodospirillum rubrum* (Falk and Walker, 1988).

### *Acetobacter pasteurianus* 386B Grows in a Defined Medium

From all carbon sources tested during the *in vitro* growth experiments, *A. pasteurianus* 386B was only able to grow on a defined medium containing lactic acid as the sole carbon source added. Thus, except for the possible need for some micronutrients, *A. pasteurianus* 386B had no specific auxotrophies and was able to form all its biomass compounds from ammonium as the sole nitrogen source, sulfate as the sole sulfur source, phosphate as the sole phosphate source, and lactic acid as the sole carbon source.

### Biosynthesis Pathways

In what follows, biosynthesis pathways are described that were included in the *A. pasteurianus* 386B GEM and for which there was genetic evidence based on the genome re-annotation. These pathways allowed to simulate the growth of *A. pasteurianus* 386B *in silico* using the defined medium conditions mentioned above. Focus is on those steps of the pathways for which there was evidence that the gene-protein-reaction associations in *A. pasteurianus* 386B were different from the reference pathways described in the literature.

#### Fatty Acid and Phospholipid Biosynthesis

The major fatty acids in bacterial cells of the genus *Acetobacter* are *cis*-vaccenic acid (C18:1), palmitic acid (C16:0), and stearic acid (C18:0) (Yamada et al., 1981). Further, the presence of a detectable amount of myristic acid (C14:0) distinguishes the genus *Acetobacter* from the genus *Gluconobacter* (Yamada et al., 1981). The fatty acid biosynthesis pathway for saturated fatty acids in *A. pasteurianus* 386B was similar to the one described in *E. coli* (Janßen and Steinbüchel, 2014). For unsaturated fatty acid biosynthesis, the FabA (EC 4.2.1.59/5.3.3.14) and FabB (EC 2.3.1.41) enzymes are critical for their formation in *E. coli* (Feng and Cronan, 2009). Although *cis*-vaccenic acid (C18:1) was the major fatty acid in the fatty acid profile of *A. pasteurianus*, the genes encoding FabA and FabB could not be found in the *A. pasteurianus* 386B genome. Even though unsaturated fatty acid biosynthesis in *A. pasteurianus* 386B seemed to be unclear, reactions of the FabA/FabB pathway were nonetheless added to the model (Supplementary Figure S5). As each fatty acid elongation cycle can be described by a stoichiometric reaction (Janßen and Steinbüchel, 2014), lumped reactions were added to the GEM to describe the formation of myristic acid (C14:0), palmitic acid (C16:0), stearic acid (C18:0) and *cis*-vaccenic acid (C18:1).

Bacterial cell membranes are composed of amphiphilic lipids, most commonly glycerophospholipids (Sohlenkamp and Geiger, 2016). Here, the phospholipid biosynthesis pathway of *Sinorhizobium meliloti* was taken as a reference (Geiger et al., 2013). In *A. pasteurianus* 386B, glycerol 3-phosphate could be produced by a quinone-dependent (EC 1.1.5.3) or NAD(P)^+^-dependent (EC 1.1.1.94) glycerol-3-phosphate dehydrogenase. The *A. pasteurianus* 386B genome encoded the PlsX/PlsY/PlsC system for phosphatidic acid biosynthesis, as described before (Parsons and Rock, 2013). Although long-chain acyl-acyl carrier protein (acyl-ACP) are the end-products of fatty acid biosynthesis and are initially transferred to a phosphate moiety by phosphate:acyl-ACP acyltransferase (PlsX), no generic reaction was available in MNXRef 3.0. Therefore, two reactions (EC 3.6.1.7 and EC 3.1.2.14) were added to the GEM to simulate acyl phosphate and acyl-ACP formation to represent the link between fatty acid biosynthesis and glycerophospholipid biosynthesis. Subsequent acylation of glycerol 3-phosphate is performed by the membrane-bound glycerol-3-phosphate acyltransferase (PlsY; EC 2.3.1.n3). Then, 1-acyl-glycerol-3-phosphate acyltransferase (PlsC; EC 2.3.1.51) forms phosphatidic acid (Paoletti et al., 2007; Yoshimura et al., 2007; Hara et al., 2008). *Acetobacter aceti* contains an unusually high amount of phosphatidylcholine in its membrane, attributed to its acetic acid resistance (Hanada et al., 2001). In *A. pasteurianus* 386B, the three methylation steps of the S-adenosyl-L-methionine (SAM)-dependent methylation pathway forming phosphatidylcholine from phosphatidylethanolamine were most probably catalyzed by the same enzyme, namely phosphatidylethanolamine N-methyltransferase (Pmt, EC 2.1.1.17), as the enzyme from *A. pasteurianus* 386B (encoded by the gene with locus tag APA386B_612) belonged to the same family as the one in *Rhodobacter sphaeroides* and shared high sequence identity to the Pmt enzyme in *A. aceti* (Hanada et al., 2001; Geiger et al., 2013).

#### Amino Acid Biosynthesis

**Figure 3:**
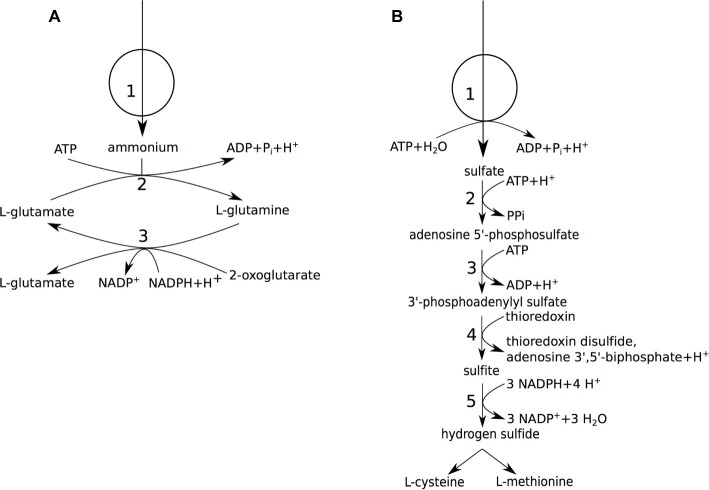
Nitrogen and sulfur metabolism of *A. pasteurianus* 386B. **(A)** Ammonium assimilation. (1) ammonium permease; (2) glutamine synthetase (EC 6.3.1.2); (3) glutamate synthase (NADPH-dependent) (EC 1.4.1.13). **(B)** Sulfate assimilation. (1) sulfate ABC transporter; (2) sulfate adenylyltransferase (EC 2.7.7.4); (3) adenylyl-sulfate kinase (EC 2.7.1.25); (4) phosphoadenylyl-sulfate reductase (thioredoxin-dependent) (EC 1.8.4.8); (5) assimilatory sulfite reductase (NADPH-dependent) (EC 1.8.1.2).

The presence of biosynthesis pathways for proteinogenic amino acids in the *A. pasteurianus* 386B PGDB was confirmed by the growth of *A. pasteurianus* 386B in a defined medium containing ammonium as the sole nitrogen source (section “*Acetobacter pasteurianus* 386B Grows in a Defined Medium”). Two genes were retrieved that encoded potential ammonium ion channels (TC 1.A.11; APA386B_239 or APA386B_740). In addition, an ammonium assimilation pathway for *de novo* biosynthesis of proteinogenic amino acids was assumed to be present. In *E. coli*, two pathways are known for ammonium assimilation (van Heeswijk et al., 2013), one occurs *via* a NADP^+^-dependent glutamate dehydrogenase (EC 1.4.1.4) that allows to form L-glutamate directly from 2-oxoglutarate and ammonium, the other *via* the formation of L-glutamate and L-glutamine through a cycle of reactions involving glutamine synthetase (EC 6.3.1.2) and glutamate synthase (EC 1.4.1.13), which produces a net amount of L-glutamate. Only the latter pathway was found in the *A. pasteurianus* 386B genome (Figure 3A; Supplementary Figure S6).

A number of pyridoxal phosphate-dependent aminotransferases were identified based on the genome annotation. These were involved in the formation of L-aspartate and L-alanine and were assigned to specific reactions based on their presence in different orthogroups. Aspartate aminotransferase (EC 2.6.1.1) was linked to three genes, likely encoding this enzyme (APA386B_861, APA386B_862, and APA386B_942). These were not predicted to be homologous to the *E. coli* aspartate aminotransferase gene (b0928, *aspC*) but shared considerable sequence identity with curated SwissProt sequences from other bacteria with identical function (>50% identity). The gene encoding glutamate-pyruvate aminotransferase (EC 2.6.1.2; APA386B_991) was a predicted ortholog of the gene in *E. coli* (b2379, *alaC*). Finally, the genes encoding aspartate 4-decarboxylase (EC 4.1.1.12; APA386B_482, APA386B_1928) had no *E. coli* homolog but were related to a bifunctional aspartate aminotransferase and aspartate 4-decarboxylase of *Comamonas testosteroni*. However, the enzyme characterized had only a minor activity as an aminotransferase (Wang and Lee, 2007). Thus, the *A. pasteurianus* 386B genes were only associated to the reaction catalyzed by aspartate 4-decarboxylase.

In the biosynthesis pathways of L-valine, L-leucine, and L-isoleucine, a bifunctional enzyme (EC 2.2.1.6) catalyzes the formation of (2S)-2-acetolactate and (S)-2-hydroxy-2-ethyl-3-oxobutanoate, the latter metabolite only involved in L-isoleucine biosynthesis (Barak and Chipman, 2012). The *A. pasteurianus* 386B genome contained three genes, which likely encode the enzymes for these reactions. The genes with locus tags APA386B_835 and APA386B_836 were most likely involved in amino acid biosynthesis, since these genes each had an *E. coli* ortholog encoding subunits of a bifunctional acetolactate synthase/acetohydroxybutanoate synthase enzyme complex (*ilvI*, *ilvH*). However, the gene with locus tag APA386B_1863 was closely related to two “catabolic” acetolactate synthases, identified in *Klebsiella pneumoniae* and *Lactococcus lactis* (Peng et al., 1992; Snoep et al., 1992). This evidence, combined with the fact that the neighboring gene APA386B_1862 was annotated as encoding an acetolactate decarboxylase, forming acetoin from (2S)-2-acetolactate, could be an indication that the physiological role of the APA386B_1863-encoded enzyme would only be related to acetoin formation. Finally, the branched-chain amino acid aminotransferase (EC 2.6.1.42/2.6.1.6; APA386B_1001) could be involved in the last step of the formation of these amino acids.

The aromatic amino acids L-phenylalanine, L-tyrosine, and L-tryptophan share a common initial pathway, which produces chorismate from erythrose 4-phosphate (Yang and Pittard, 2008). Comparative genomics revealed that the gene with locus tag APA386B_1330, encoding a dehydroquinate dehydratase (EC 4.2.1.10), had an ortholog in *G. oxydans* 621H (locus tag GOX0437) that has been identified as encoding a periplasmic enzyme involved in the oxidation of quinate (Adachi et al., 2008). The cytoplasmic dehydroquinate dehydratase of *G. oxydans* 621H (locus tag GOX1351) could not be found in the *A. pasteurianus* 386B genome. In the last reaction step of the biosynthesis of L-tyrosine and L-phenylalanine, an amino group transfer occurs with glutamate as amino group donor. In *E. coli*, three enzymes have been identified that could catalyze this reaction, namely the aromatic aminotransferase (TyrB), the aspartate aminotransferase (AspC), and the branched-chain amino acid aminotransferase (IlvE), whereby the latter is only involved in the biosynthesis of L-phenylalanine (Yang and Pittard, 2008). In *A. pasteurianus* 386B, only an ortholog of the gene encoding IlvE was found (APA386B_1001). Thus, the L-tyrosine-forming reaction was tentatively associated to the aspartate aminotransferases encoded in the genome (APA386B_861, APA386B_862, and APA386B_942).

Since *A. pasteurianus* 386B was able to grow in a defined medium containing sulfate as the sole sulfur source, sulfate assimilation was assumed (Figure 3B). Indeed, import of sulfate ions could occur *via* a sulfate permease (TC 2.A.53) as well as a probable ortholog of the *E. coli cysPUWA* ABC sulfate transporter. However, since the stoichiometry of the anion:H^+^ symporter is not known in *Acetobacter*, only the ABC transporter was added to the *A. pasteurianus* 386B GEM. The enzymes of the assimilatory sulfate reduction pathway, which reduce sulfate to hydrogen sulfide, were encoded in the genome. One exception was the flavoprotein subunit of sulfite reductase (EC 1.8.1.2), which was not found in the genome re-annotation. Whereas L-methionine is produced by the transsulfuration pathway in *E. coli*, only cystathionine 𝛽-lyase (EC 4.4.1.8), but not homoserine *O*-succinyltransferase (EC 2.3.1.46), could be identified in *A. pasteurianus* 386B. In *Corynebacterium glutamicum*, next to the transsulfuration pathway, a direct sulfhydrylation pathway is present that produces L-methionine, of which the key enzyme is *O*-acetylhomoserine sulfhydrylase (EC 2.5.1.49) (Hwang et al., 2002). The gene with locus tag APA386B_753 was annotated as encoding a putative bifunctional *O*-acetylhomoserine sulfhydrylase (EC 2.5.1.49)/*O*-succinylhomoserine sulfhydrylase (EC 2.5.1.48). However, the presence of a homoserine *O*-acetyltransferase (E.C. 2.3.1.31; APA386B_2138) upstream in the direct sulfhydrylation pathway gave additional evidence to annotate the gene APA386B_753 as encoding an *O*-acetylhomoserine sulfhydrylase (E.C. 2.5.1.49). Since this pathway was complete, the direct sulfhydrylation pathway was added to the *A. pasteurianus* 386B GEM instead of the transsulfuration pathway. Finally, L-methionine could be synthesized from L-homocysteine by methionine synthase (EC 2.1.1.13). SAM could be formed from L-methionine by methionine adenosyltransferase (EC 2.5.1.6). This reaction is part of the SAM cycle, which links L-methionine biosynthesis to glycerophospholipid biosynthesis, and involves the key reaction catalyzed by adenosylhomocysteinase (EC 3.3.1.1) (Reddy et al., 2008).

#### Nucleotide Biosynthesis

Based on the *A. pasteurianus* 386B PGDB, the nucleotide biosynthesis pathways were curated and added to the *A. pasteurianus* 386B GEM (Supplementary Figure S7). A broad-substrate-range nucleoside diphosphate kinase (EC 2.7.4.6) was present in the genome of *A. pasteurianus* 386B that could phosphorylate different nucleoside diphosphate acceptors, using ATP as phosphate donor, to form the respective nucleoside triphosphate products (Armenta-Medina et al., 2014). Similarly, a broad-substrate-range thioredoxin-dependent ribonucleoside-diphosphate reductase (EC 1.17.4.1) could reduce ribonucleoside diphosphate acceptors to their respective deoxyribonucleoside diphosphate forms. The thioredoxin molecule may be recycled by an NADP^+^-dependent thioredoxin reductase (EC 1.8.1.9). The precursor for purine biosynthesis is 5-phospho-α-D-ribose 1-phosphate (PRPP), which is also the starting point of histidine biosynthesis and involved in tryptophan biosynthesis (Kilstrup et al., 2005). From PRPP, a linear pathway was obtained to produce inosine monophosphate, from which adenosine monophosphate (AMP) or guanosine monophosphate (GMP) could be produced. Two additional adenosine salvage reactions were identified, which were related to side-products formed in other pathways. First, sulfate reduction to hydrogen sulfide could yield adenosine 3′,5′-bisphosphate as a by-product of the initial adenylation of sulfate by ATP. This compound could be recycled to AMP by 3′,5′-bisphosphate nucleotidase (EC 3.1.3.7), which was previously not annotated in the *A. pasteurianus* 386B genome. Second, the SAM cycle could produce adenosine, which could be recycled to AMP by adenosine kinase (EC 2.7.1.20).

Uridine diphosphate (UDP) production is the result of a linear pathway, in which aspartate is the substrate for pyrimidine biosynthesis and which could be converted into cytidine triphosphate (CTP) *via* CTP synthase (EC 6.3.4.2) or deoxythymidine triphosphate (dTTP) by a dedicated pathway (Kilstrup et al., 2005). Cytidine monophosphate (CMP) could be salvaged by cytidylate kinase (EC 2.7.4.25), as CMP was a side-product from phospholipid and lipopolysaccharide (LPS) biosynthesis. Consecutive cytidine diphosphate (CDP) reduction by ribonucleoside-diphosphate reductase and phosphorylation by nucleoside diphosphate kinase could produce dCTP.

#### Peptidoglycan and Lipopolysaccharide Biosynthesis

Biosynthesis pathways of membrane components in *E. coli* were used as a reference for their reconstruction in *A. pasteurianus* 386B (Supplementary Figures S8, S9). In general, the biosynthesis of peptidoglycan starts with the formation of glucosamine 6-phosphate by glutamine-fructose-6-phosphate transaminase (EC 2.6.1.16), which is subsequently biochemically activated to form UDP-*N*-acetylglucosamine (UDP-GlcNAc). UDP-GlcNAc is subsequently converted into UDP-*N*-acetylmuramic acid (UDP-MurNAc) and used as a basis to attach the first alanine residue by UDP-*N*-acetylmuramate L-alanine ligase (EC 6.3.2.8), which was previously not annotated in the *A. pasteurianus* 386B genome. In the next reactions, D-glutamate, meso-diaminopimelate, and D-alanyl-D-alanine are successively attached to UDP-MurNAc (Barreteau et al., 2008). Finally, another molecule of UDP-GlcNAc is linked to UDP-MurNAc to form the peptidoglycan monomer (Vollmer et al., 2008). This last step was included in the macromolecule reaction forming peptidoglycan (Supplementary Figure S1). Whereas it involves *in vivo* the linkage of the peptidoglycan precursor to undecaprenyl-phosphate (Typas et al., 2012), a growing peptidoglycan chain requires the recycling of undecaprenyl-phosphate, so its biosynthesis was not included in the model.

For lipopolysaccharide biosynthesis, UDP-GlcNAc is an important precursor to form (Kdo)_2_-lipid A *via* the Raetz pathway (Whitfield and Trent, 2014). In *A. pasteurianus* 386B, UDP-2,3-diacylglucosamine pyrophosphatase (EC 3.6.1.54) could form the intermediary lipid X. In contrast to *E. coli* that has the *lpxH* gene, in *A. pasteurianus* 386B and other α-proteobacteria this enzyme is encoded by the *lpxI* gene, which has a different reaction mechanism (Metzger and Raetz, 2010). Lipid X could be converted into lipid IV and two molecules of CMP-ketodeoxyoctonate (CMP-Kdo) could be transferred to lipid IV by a bifunctional 3-deoxy-D-manno-octulosonic-acid transferase (EC 2.4.99.12 and EC 2.4.99.13). Finally, two fatty acyl chains could be added to form (Kdo)_2_-lipid A. Whereas *E. coli* has two distinct enzymes encoded by *lpxL* and *lpxM*, one for each fatty acyl chain transfer, of which *lpxM* is not required for growth (Raetz et al., 2007), *A. pasteurianus* 386B possessed only one putative ortholog, APA386B_2689, which was most similar to *lpxL*. Subsequently, a core oligosaccharide unit, of which the composition may differ between microorganisms, is synthesized and attached to lipid A (Raetz and Whitfield, 2002). Here, the pathway present in *A. pasteurianus* 386B diverged from the one known in *E. coli*, since only four out of 10 described enzymatic reactions to synthesize the *E. coli* core oligosaccharide could be linked to putative *A. pasteurianus* 386B orthologs. Furthermore, two enzymes were missing in the pathway to produce ADP-L-glycero-D-manno-heptose. These reactions were nonetheless added to the model to allow for biomass formation (Supplementary Figure S9). Genes encoding enzymes to form the LPS precursors UDP-glucose and UDP-galactose were present, including the genes encoding phosphoglucomutase (EC 5.4.2.2), UTP-glucose-1-phosphate uridylyltransferase (EC 2.7.7.9), and UDP-glucose 4-epimerase (EC 5.1.3.2). Biosynthesis reactions for the formation of lipoprotein were not added to the *A. pasteurianus* 386B GEM, since there was no information about its abundance in the biomass composition data used.

### Model Validation

*In vitro* and *in silico* growth experiments were compared to validate the *A. pasteurianus* 386B GEM (Table 1). This strain has been routinely cultivated in a cocoa pulp simulation medium containing lactic acid, ethanol, and mannitol as the main carbon sources (Lefeber et al., 2010; Moens et al., 2014). Here, the carbon sources were tested separately to assess their influence on the predicted flux distribution in isolation. For all tested carbon sources, periplasmic proton exchange was necessary to obtain growth *in silico*.

**Table 1:** *In vitro* growth experiments and *in silico* predicted specific growth rate using different carbon sources.

Carbon source	*In vitro* growth	*In silico* specific growth rate (h^−1^)
D-glucose	No	0.66
D-mannitol	No	0.66
Glycerol	No	1.12
Lactic acid	Yes	0.85
Ethanol	No	0.00
Acetic acid	No	0.00

Growth of *A. pasteurianus* 386B on lactic acid as the sole carbon source was found *in silico* as well as *in vitro*. CO_2_ and H_2_O were the sole metabolites secreted by the model, which was in accordance to the experimental results obtained with another *A. pasteurianus* strain (Adler et al., 2014). *A. pasteurianus* 386B was not able to grow on ethanol as the sole carbon source, which confirms the growth characteristics of *A. pasteurianus* and is in contrast to other species of the genus *Acetobacter* (Cleenwerck et al., 2008). This result was also obtained when acetate was the sole carbon source. These results are probably related to the absence of genes encoding enzymes of the glyoxylate cycle in the *A. pasteurianus* 386B genome, since this cycle is known to be crucial for growth on C2 sources such as ethanol and acetate (Illeghems et al., 2013).

In contrast to the results of the *in vitro* growth experiments, *in silico* growth was possible on D-glucose and D-mannitol as the sole carbon sources. In both cases, FBA predicted their catabolism by the pentose phosphate pathway, leading to the formation of fructose-6-phosphate and glyceraldehyde-3-phosphate. The latter was further catabolized in the lower part of the Embden-Meyerhof-Parnas (EMP) pathway. Due to the absence of a phosphofructokinase enzyme, fructose-6-phosphate was converted into glucose-6-phosphate, leading to a reaction flux cycle involving the pentose phosphate pathway and the upper part of the EMP pathway. A high NADPH+H^+^ production flux had to be balanced to allow growth, which was mainly performed by a proton-translocating NAD(P)^+^ transhydrogenase. The contrasting results obtained for D-glucose and D-mannitol might be explained by the fact that no specific transporter could be identified for these metabolites or their oxidation products. In general, functional annotation of transporters is difficult because a limited number of transporters have been functionally characterized (Gelfand and Rodionov, 2008). It is therefore possible that D-glucose and D-mannitol are almost exclusively oxidized in the periplasm, forming D-gluconate and D-fructose, respectively (Moens et al., 2014). In addition, the occurrence of a reaction flux cycle in the FBA prediction could be an indication of the incapability of sugar catabolism to sustain growth of *A. pasteurianus* 386B.

A similar discrepancy for *in vitro* and *in silico* growth was found for glycerol. The predicted specific growth rate on glycerol was the highest of all carbon sources tested, but *in silico* growth was only possible if the reaction catalyzed by the aerobic glycerol-3-phosphate dehydrogenase (EC 1.1.5.3) was defined as being reversible. Here, a glycerol facilitator could be identified in the genome of *A. pasteurianus* 386B. No apparent reason for the absence of *in vitro* growth could be identified in the flux distribution. However, the assumption of the presence of a catabolic glycerol-3-phosphate dehydrogenase may be faulty or glycerol catabolism was not efficient enough for the bacterial cells to grow on glycerol as the sole carbon source.

### Evaluation of the Manual Curation Process and *Acetobacter pasteurianus* 386B GEM Properties

**Figure 4:**
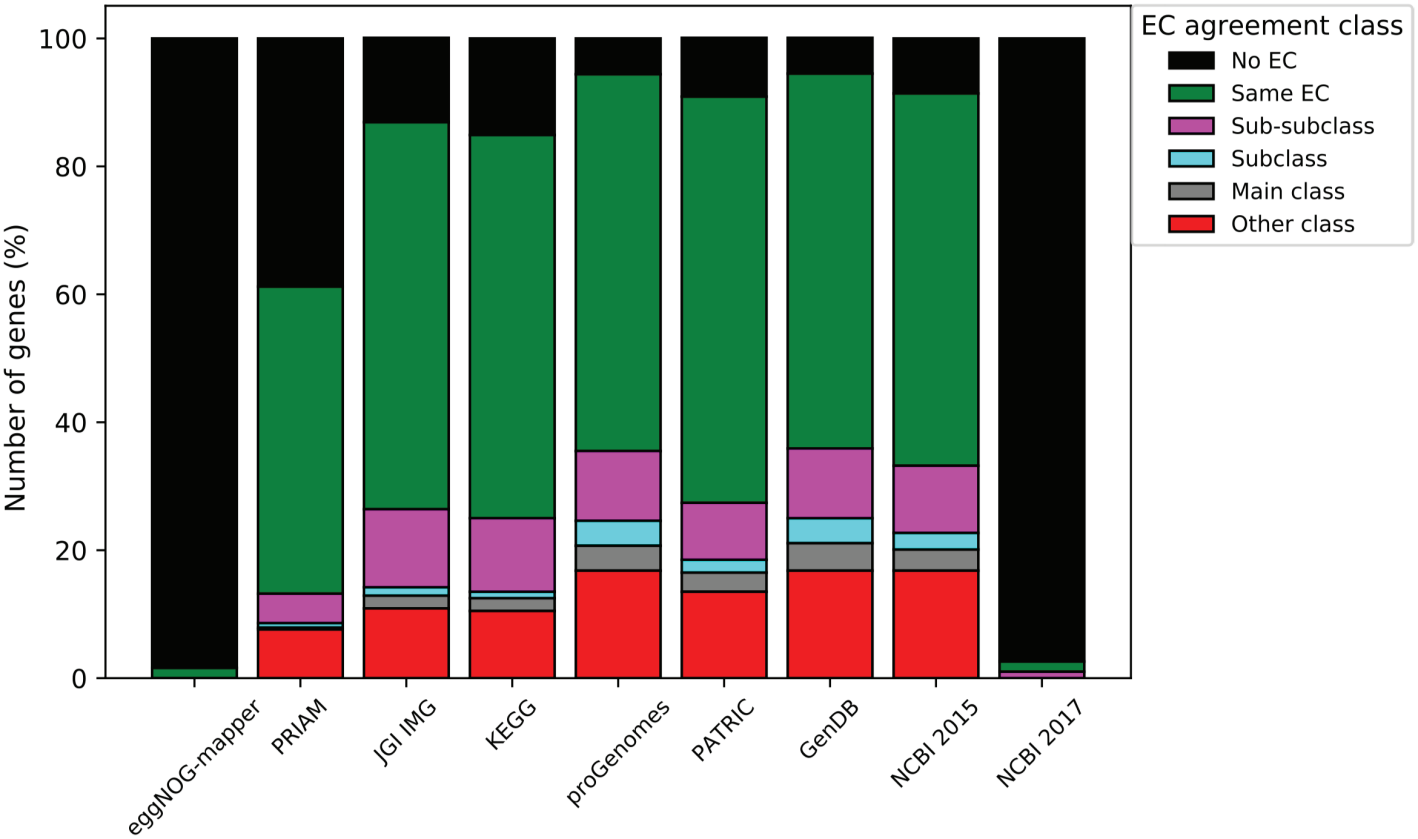
Comparison of EC number annotations of genes in the *A. pasteurianus* 386B GEM with EC numbers present in other annotation sources. The levels of agreement between annotated EC numbers were classified in a sub-subclass (three first numbers identical), subclass (two first numbers identical), main class (first number identical), and other class (different first number), according to the EC classification system. The number of genes classified in each class is shown for each of the annotation sources considered.

Manual curation of GPR associations in the *A. pasteurianus* 386B GEM was performed based on different information sources. Hereto, EC numbers were used, as these provide a defined classification of enzymatic reactions. However, GPR associations may be complex and it is thus expected that curation will reveal inconsistencies in the functional annotations provided by the different annotation sources. For genes linked to reactions in the GEM, the differences between the manually curated EC numbers and the EC numbers provided by each annotation source were compared (Figure 4). Some annotation sources, such as eggNOG-mapper and NCBI 2017, did not provide much EC information. EggNOG-mapper annotations are characterized by long annotation notes, mostly descriptive and evading EC classification. For the NCBI annotation, the outcome is related to the RefSeq annotation version used that apparently only contained very few EC numbers. Although overall the EC numbers in the *A. pasteurianus* 386B GEM corresponded to the ones in the different annotation sources, differences were apparent at the main class or sub-subclass EC classification. The former indicated, for example, the occurrence of multi-functional enzymes, linked to more than one reaction, with EC numbers belonging to different classes. Also, proton-translocating enzymes were recently re-classified in a new EC class of translocases (EC 7). The latter highlighted the subtleties of the manual curation process in defining the substrate and co-factor specificities.

The properties of the resulting *A. pasteurianus* 386B GEM, the curated *A. pasteurianus* 386B PGDB, and the models of the automatic reconstruction tools are summarized in Table 2. Compartmentalization differed between the different reconstructions, probably due to a difference in ways to represent a GEM from Gram-negative bacteria. However, since compartmentalization represents important physiological barriers, it most likely influences the GEM simulation results. As could be expected, the number of genes in the automatically reconstructed models was higher than for the *A. pasteurianus* 386B GEM presented in the current study, as this reconstruction was performed manually and because some pathways were deliberately not included, such as the co-factor biosynthesis pathways. This latter decision was taken based on the assumption that the biosynthesis and degradation fluxes of the reaction co-factors were small compared to their involvement in the metabolic redox reactions. In contrast, the number of orphan reactions was much lower for the *A. pasteurianus* 386B GEM compared to the other models, providing causal links between reactions and enzyme-encoding genes in the genome. Also, the number of dead-end metabolites was low compared to the automatic reconstructions. Although dead-end metabolites could be an indication of redundancy in the reconstruction, their presence may also reflect the uncertainty in the reactants and products of the different enzymatic reactions in the models, thus providing a compendium of possible reactants and products, as for example in the curated *A. pasteurianus* 386B PGDB.

**Table 2:** Properties of currently available reconstructed genome-scale metabolic models of *A. pasteurianus* 386B.

Property	iAp386B454	CarveMe	KBase	MNX	PGDB	PGDB_curated
Compartments	2	3	2	3	0	0
Pathways	NA	NA	NA	NA	294	213
Genes	454	611	697	820	723	829
Reactions	322	1,424	1,061	2,712	1,514	1818
Exchange reactions (% of reactions)	17 (5%)	135 (9%)	98 (9%)	245 (9%)	0 (0%)	0 (0%)
Irreversible reactions (% of reactions)	135 (42%)	946 (66%)	477 (45%)	1,451 (54%)	1,052 (69%)	1,493 (82%)
Orphan reactions (% of reactions)	32 (10%)	473 (33%)	107 (10%)	778 (29%)	468 (30%)	308 (16%)
Metabolites	296	1,078	1,026	1,535	1,608	1,759
Dead-end metabolites (% of metabolites)	2 (1%)	19 (2%)	305 (30%)	90 (6%)	701 (43%)	719 (40%)

*Curated reconstructions include iAp386B454 and PGDB_curated. Draft reconstructions include CarveMe, KBase, MNX, and PGDB (Pathway Tools). Exchange reactions represent a possible exchange of metabolites with the environment. Irreversible reactions are based on the flux bounds in the SBML file. Orphan reactions have no GPR association. Dead-end metabolites have only a single reaction partner. NA, not available*.

## Conclusion

During the genome re-annotation of *A. pasteurianus* 386B, the functional annotation of predicted enzymes and transporters was targeted, as these are critical for an accurate genome-scale metabolic network reconstruction. To improve the quality of the re-annotation, information from multiple, different annotation sources was combined, which proved to be a good strategy to guide manual curation of GPR associations. This methodology was combined with the prediction of orthogroups, using genomes of related species, which was further fine-tuned with information from the literature. Finally, using the Pathway Tools software allowed to set the annotation content in a metabolic pathway context. In this way, possible links between the different biosynthesis pathways, necessary for the biomass formation, were identified.

The re-annotated *A. pasteurianus* 386B genome was used to compile a curated GEM, named iAp386B454, containing 454 genes, 322 reactions, and 296 metabolites embedded in two cellular compartments. The GEM is available in SBML level 3 format and as a curated Pathway Tools PGDB and represents the first *in silico* genome-scale metabolic network reconstruction of a species of the genus *Acetobacter*. The reconstructed model was validated by performing growth experiments in a defined medium, which revealed that lactic acid as the sole carbon source could sustain growth of this strain.

Nevertheless, it became clear that some knowledge gaps remained, for example for the reconstruction of the biosynthesis pathways of cell constituents, especially for the cell envelope. Also, the combination of genome re-annotation and growth experiments could not resolve the presence of all metabolite transporters. The results obtained in this study will help to guide future research to close these knowledge gaps in *A. pasteurianus*.

## Data Availability Statement

The curated *A. pasteurianus* 386B PGDB of the current study can be found in the BioCyc Tier-2 repository.

## Author Contributions

RP and SW designed the study. RP performed the metabolic reconstruction and wrote the manuscript. DG and SW supervised the study, discussed the results, and edited the manuscript. BT contributed to the discussion of the results. LD contributed to the study and edited the manuscript. All authors approved the manuscript.

### Conflict of Interest

The authors declare that the research was conducted in the absence of any commercial or financial relationships that could be construed as a potential conflict of interest.

## Supplementary Material

SUPPLEMENTARY FILE S1, TABLE S1 Best blast hits of protein sequences of characterized enzymes to the *A. pasteurianus* 386B genome.

SUPPLEMENTARY FILE S1, FIGUREs S1–S9 Reactions with EC numbers of the different pathways included in the *A. pasteurianus* 386B GEM.

SUPPLEMENTARY FILE S2 Gene-protein-reaction associations of the *A. pasteurianus* 386B GEM.

SUPPLEMENTARY FILE S3 SBML file of the *A. pasteurianus* 386B GEM.

SUPPLEMENTARY FILE S4 Biomass reaction for *A. pasteurianus* 386B using a combination of genomic and literature data.
