# Supplementary material for: Genome-Scale Metabolic Reconstruction of Acetobacter pasteurianus 386B, a Candidate Functional Starter Culture for Cocoa Bean Fermentation
Source: Front Microbiol. 2019 Dec 5;10:2801. doi: 10.3389/fmicb.2019.02801 (PMC6915089; doi:10.3389/fmicb.2019.02801)
Supplement: SUPPLEMENTARY FILE S1, TABLE S1 — Best blast hits of protein sequences of characterized enzymes to the A. pasteurianus 386B genome. [file Data_Sheet_1.doc]

Supplementary Material

# Supplementary Tables

#
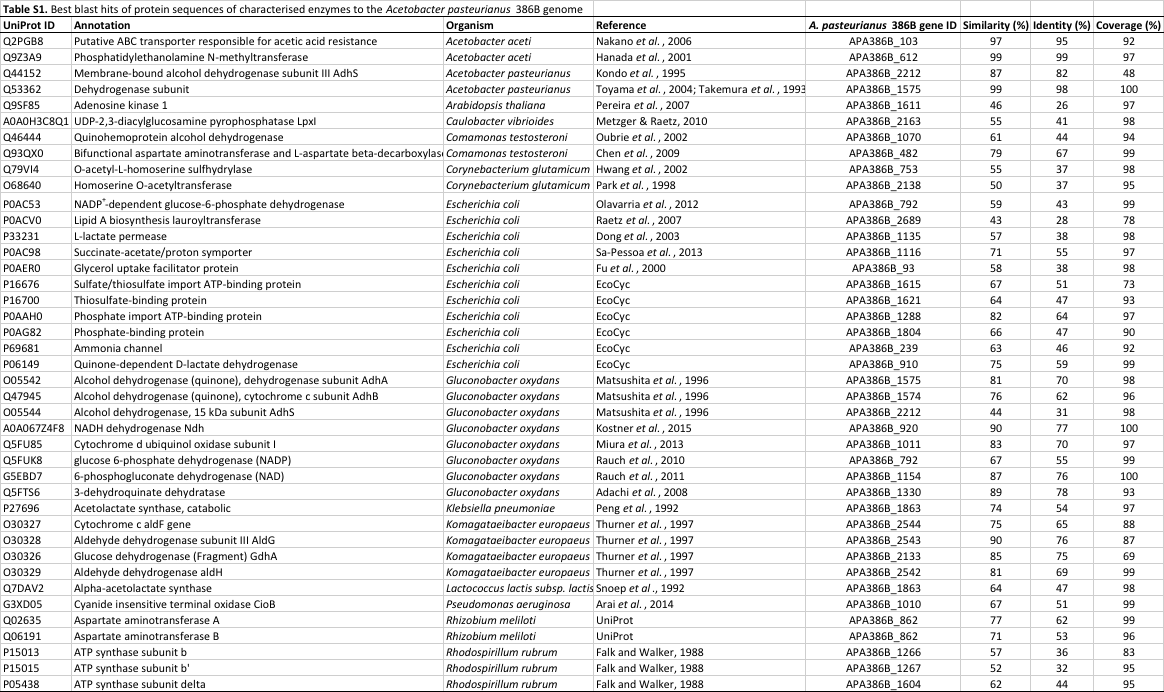


# Supplementary Figures

#
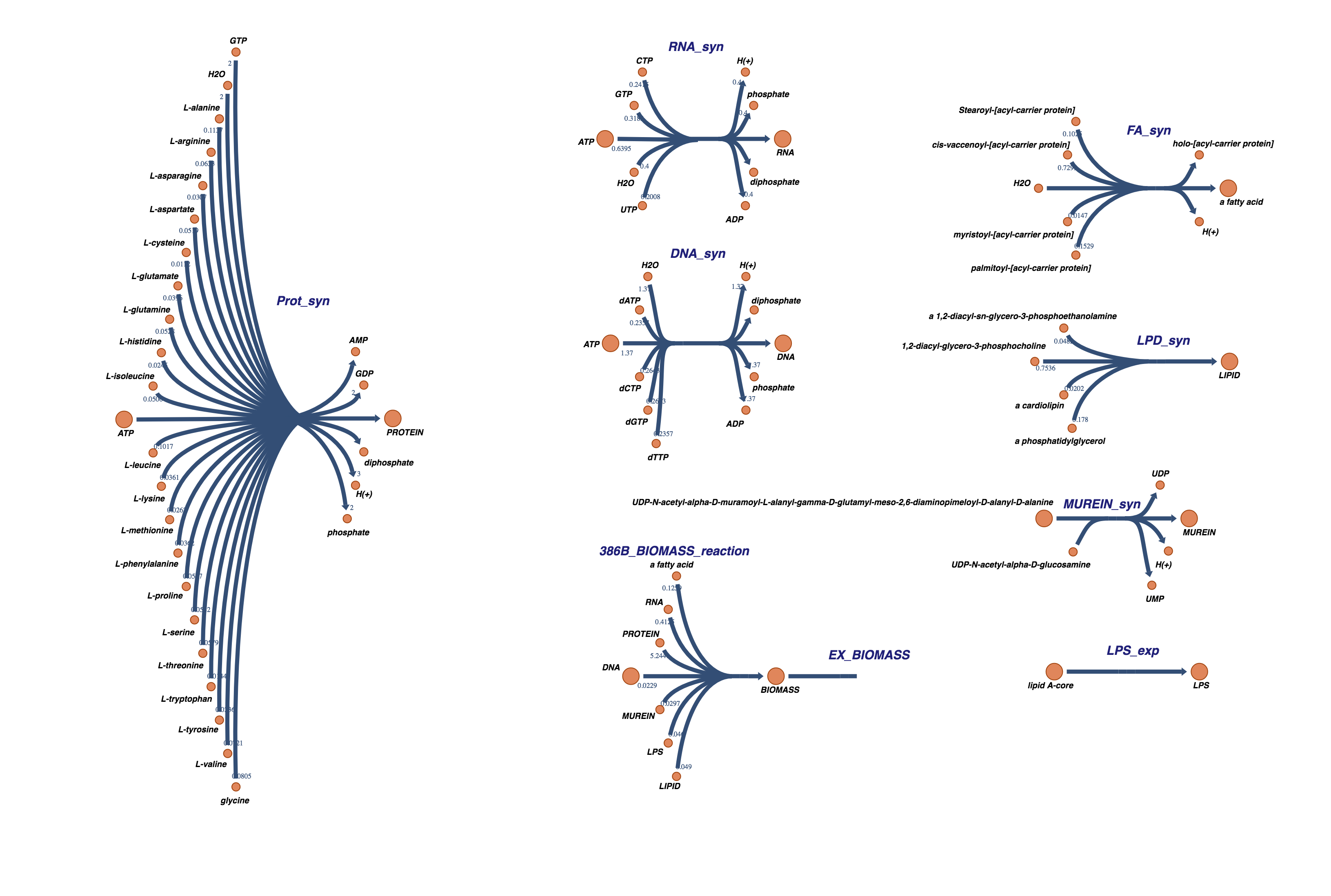


**Supplementary Figure 1.** Macromolecule biosynthesis reactions and biomass reaction as incorporated in the *Acetobacter pasteurianus* 386B GEM

#
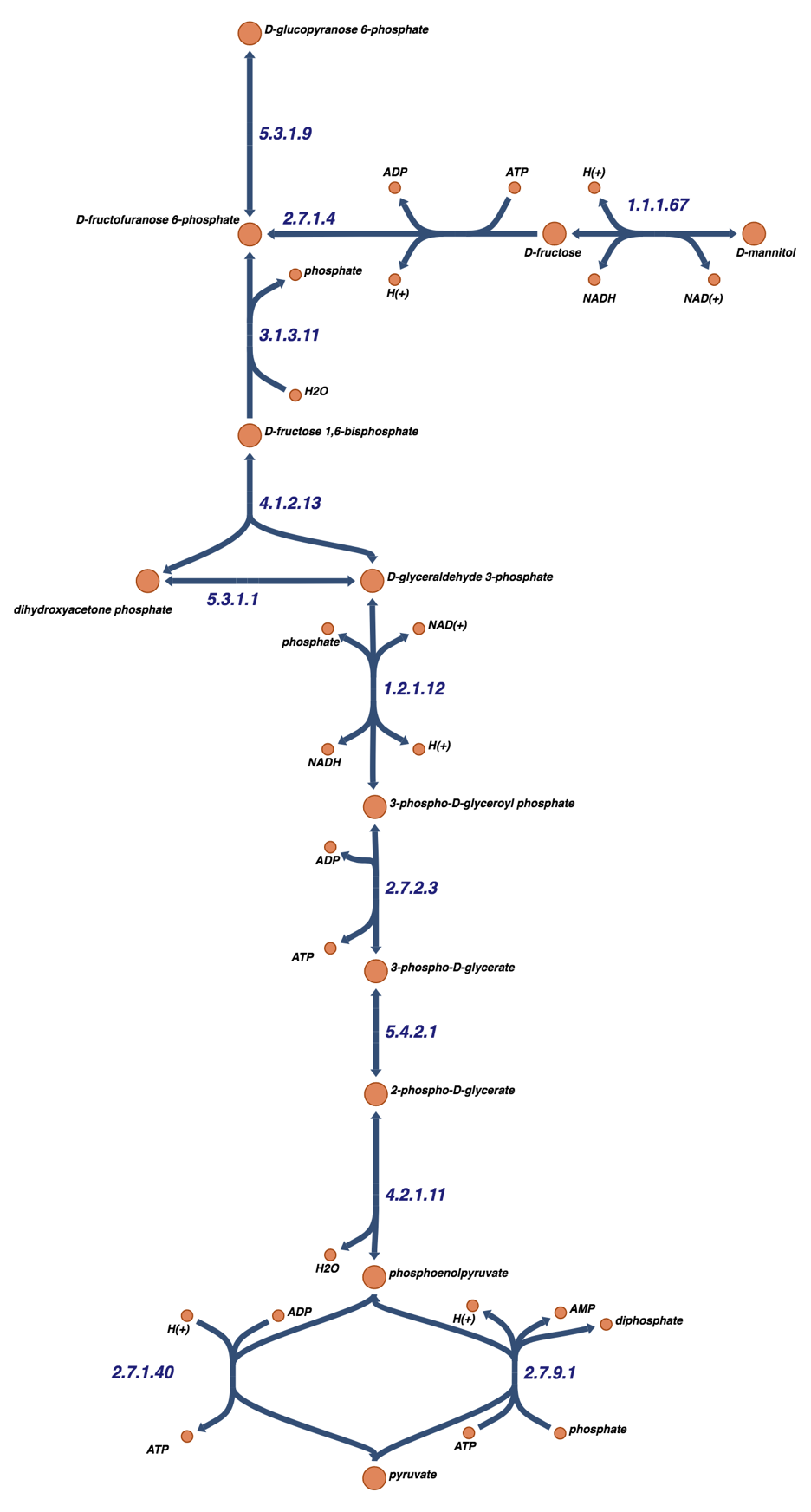


**Supplementary Figure 2.** Reactions with EC numbers of the Embden-Meyerhof-Parnas pathway as incorporated in the *Acetobacter pasteurianus* 386B GEM

#
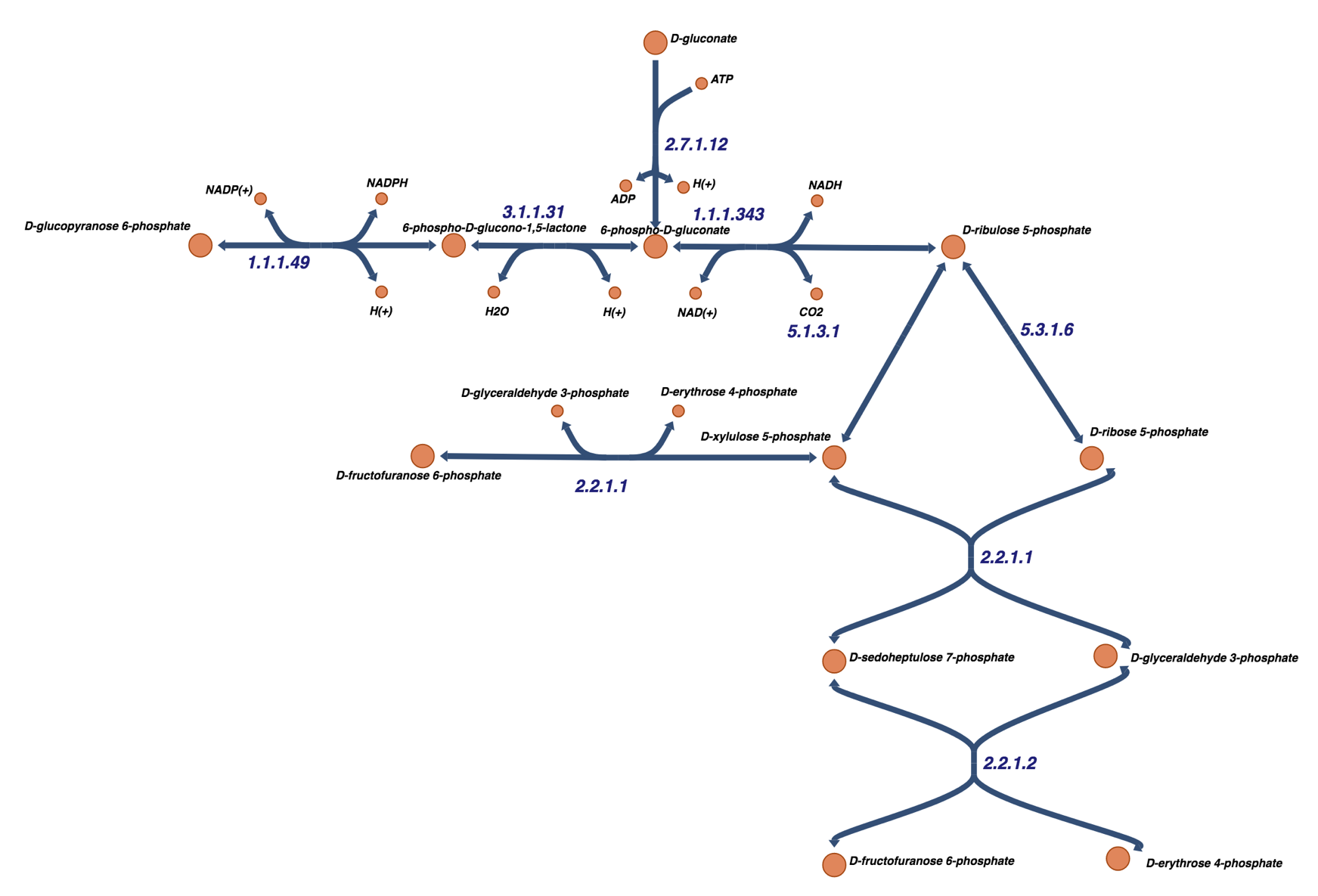


**Supplementary Figure 3.** Reactions with EC numbers of the pentose phosphate pathway as incorporated in the *Acetobacter pasteurianus* 386B GEM


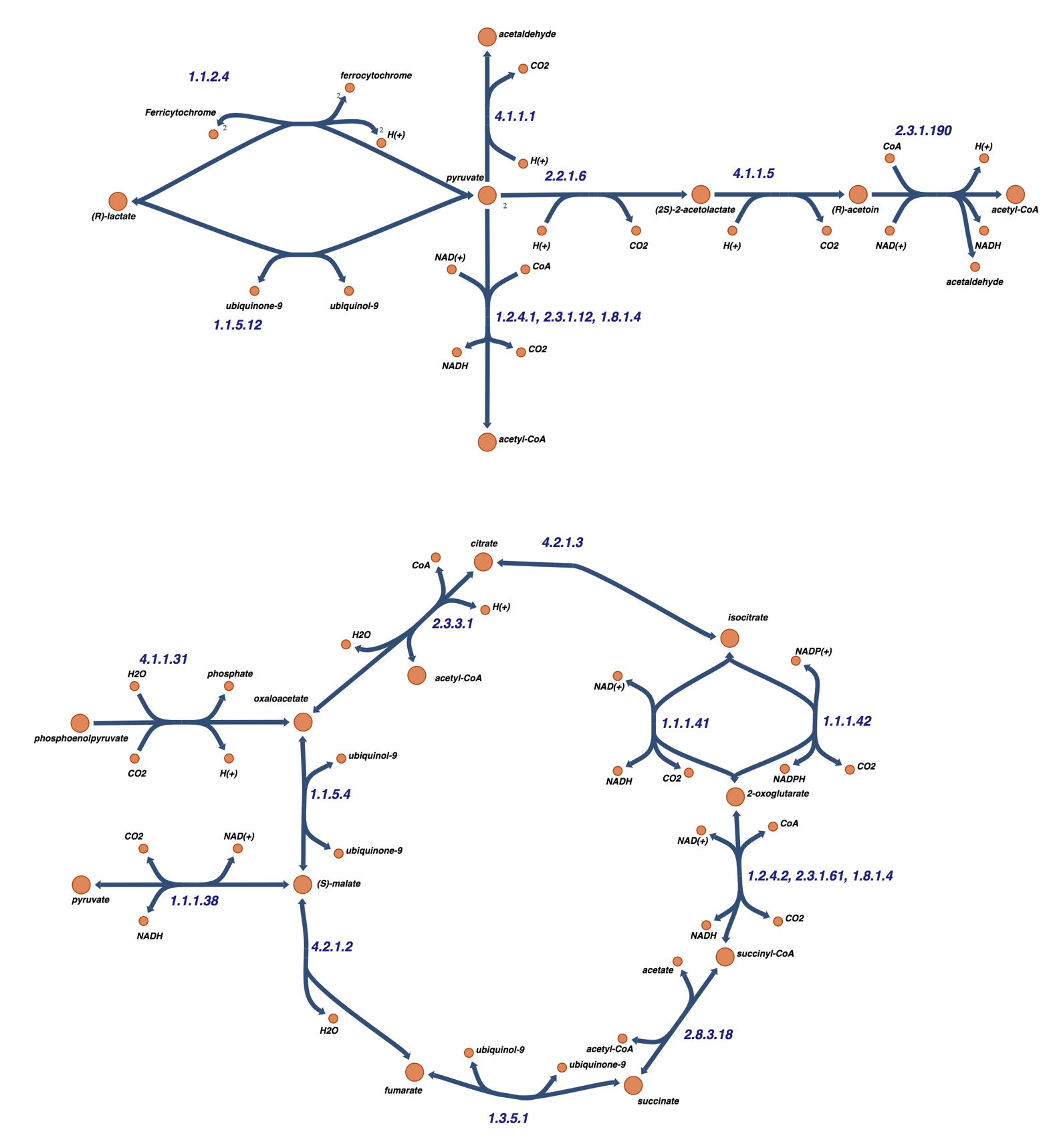


**Supplementary Figure 4.** Reactions with EC numbers of pyruvate metabolism and the tricarboxylic acid cycle as incorporated in the *Acetobacter pasteurianus* 386B GEM


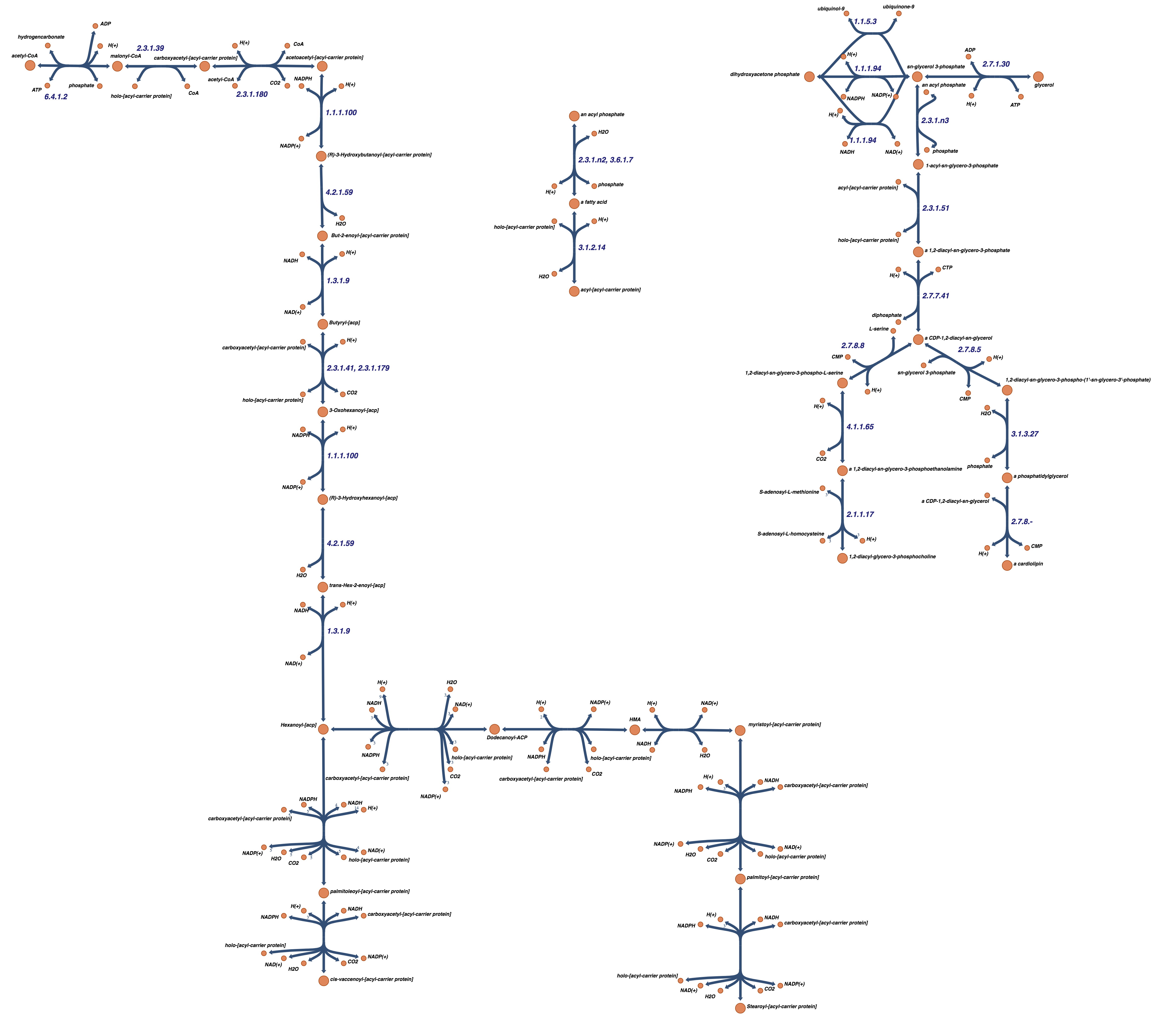


**Supplementary Figure 5.** Reactions with EC numbers of fatty acid and phospholipid biosynthesis pathways as incorporated in the *Acetobacter pasteurianus* 386B GEM


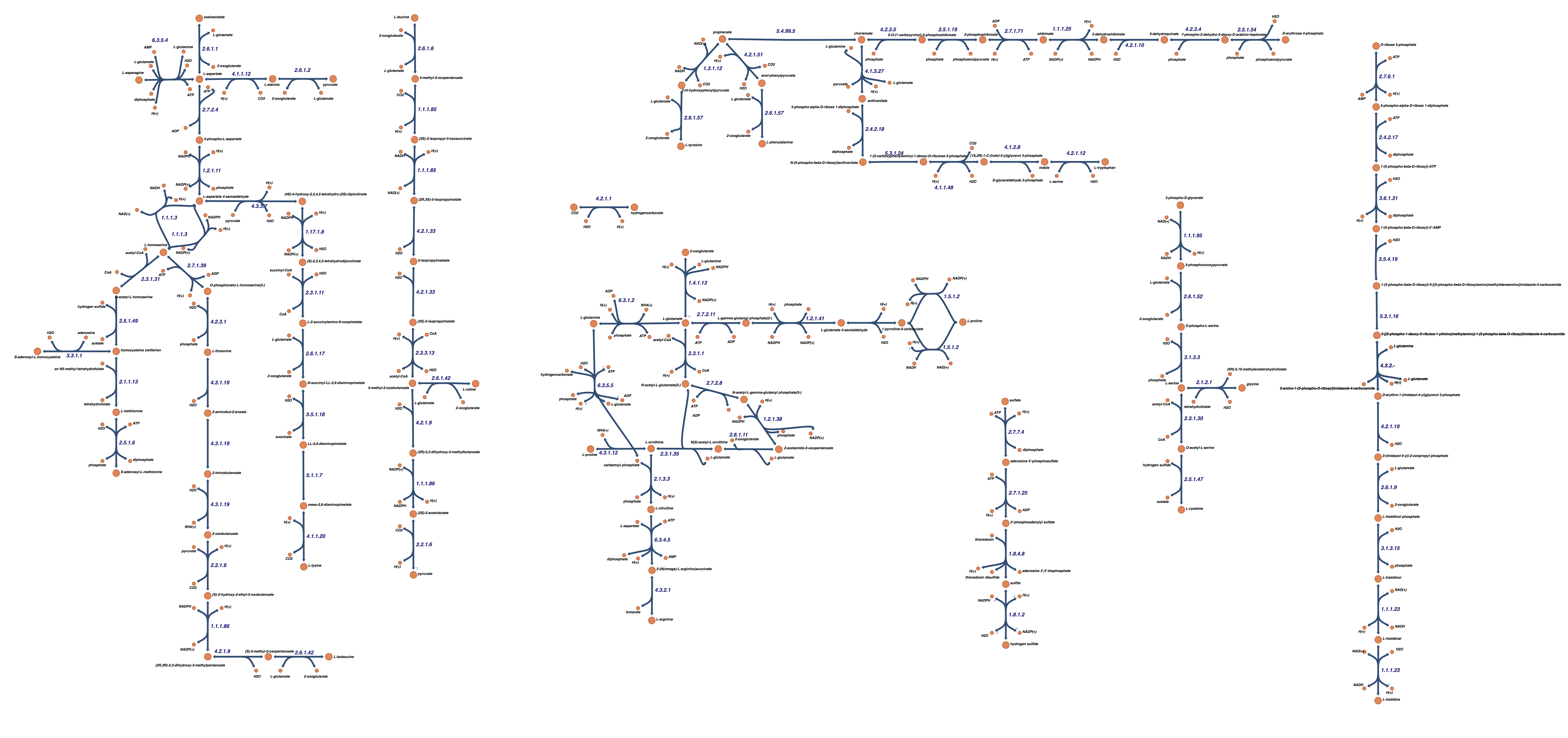


**Supplementary Figure 6.** Reactions with EC numbers of amino acid biosynthesis pathways as incorporated in the *Acetobacter pasteurianus* 386B GEM


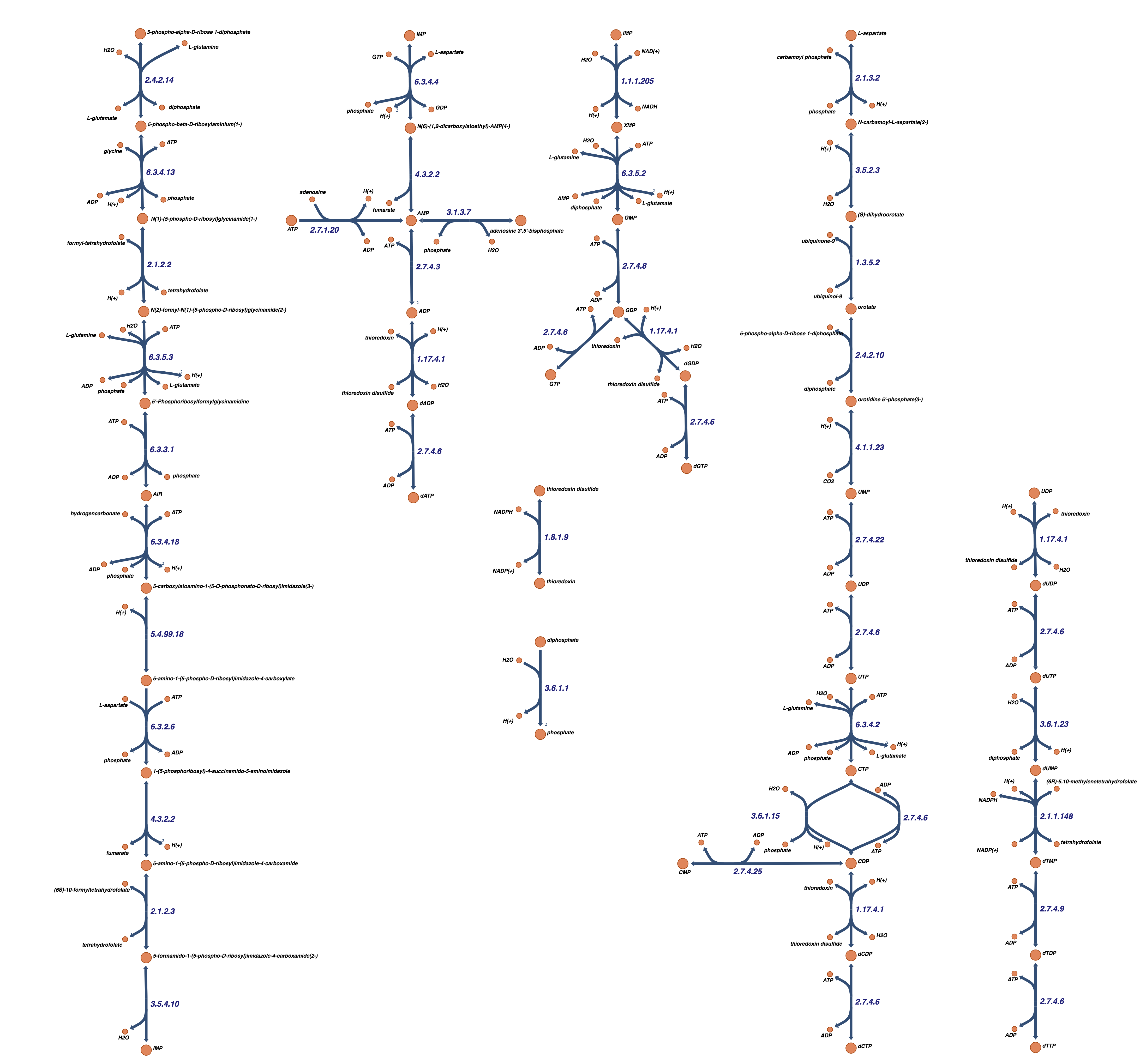


**Supplementary Figure 7.** Reactions with EC numbers of nucleotide biosynthesis pathways as incorporated in the *Acetobacter pasteurianus* 386B GEM


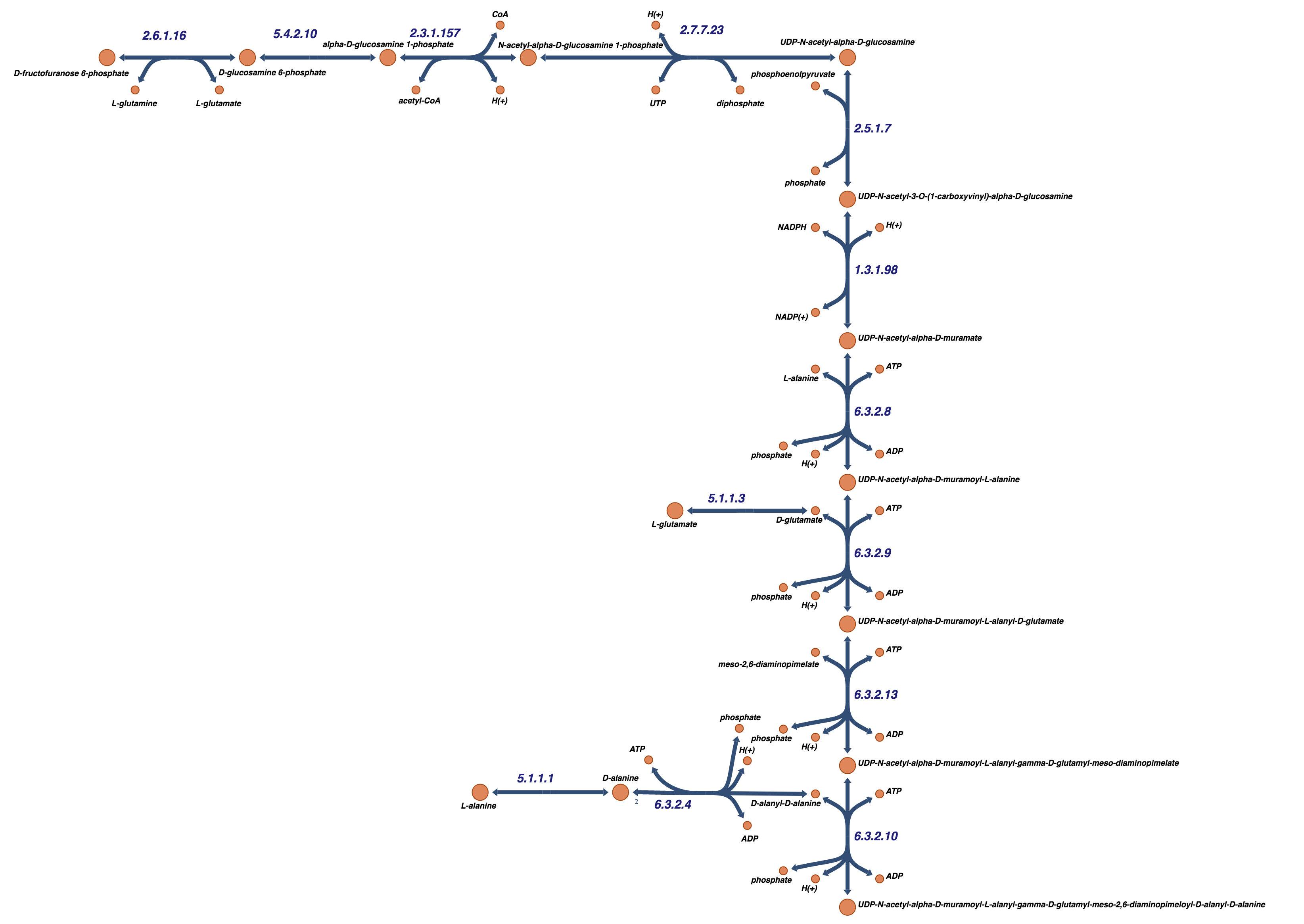


**Supplementary Figure 8.** Reactions with EC numbers of the peptidoglycan biosynthesis pathway as incorporated in the *Acetobacter pasteurianus* 386B GEM


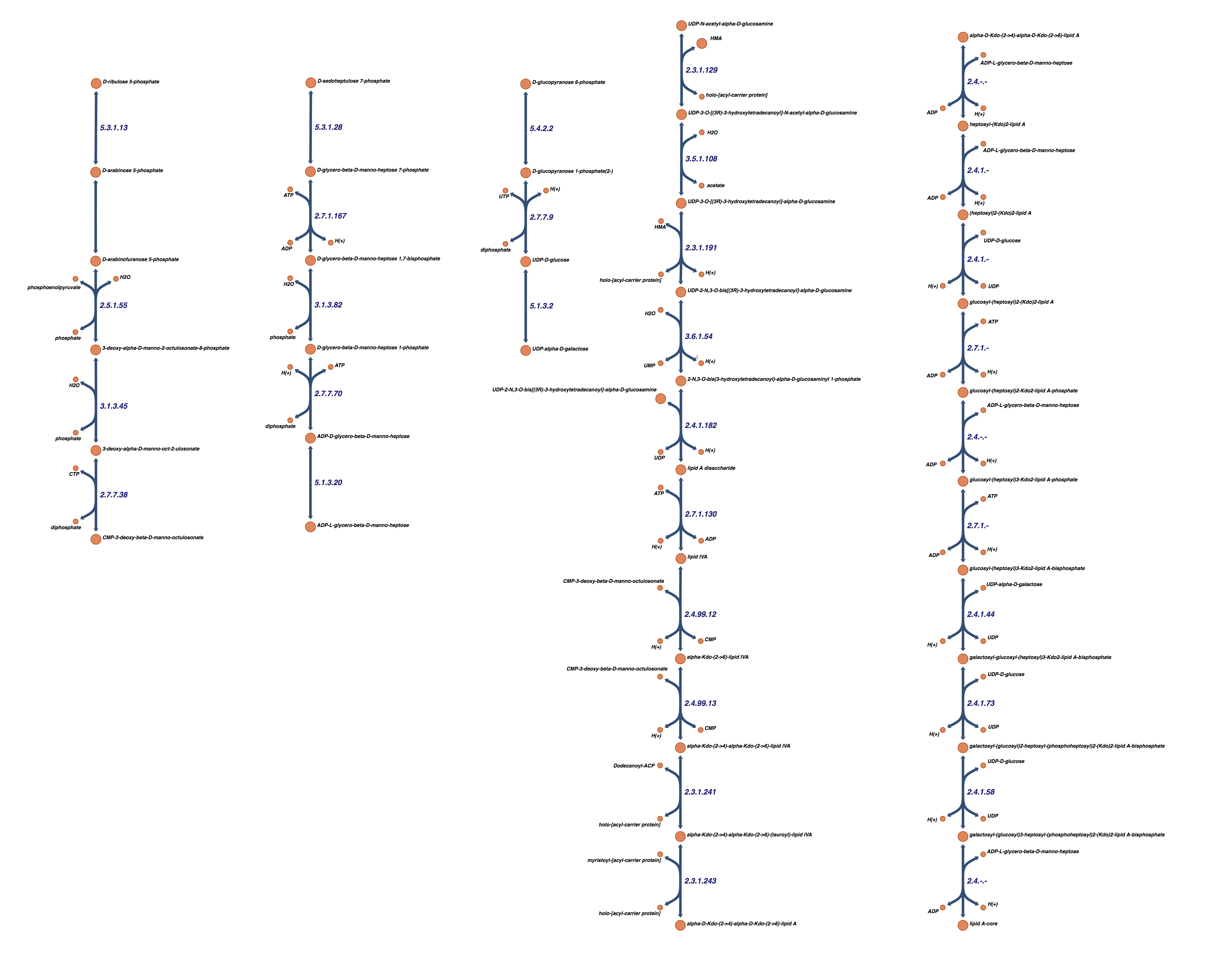


**Supplementary Figure 9.** Reactions with EC numbers of the lipopolysaccharide biosynthesis pathway as incorporated in the *Acetobacter pasteurianus* 386B GEM
